# Iatrogenic Creutzfeldt-Jakob disease with Amyloid-β pathology: an international study

**DOI:** 10.1186/s40478-017-0503-z

**Published:** 2018-01-08

**Authors:** Ignazio Cali, Mark L. Cohen, Stéphane Haїk, Piero Parchi, Giorgio Giaccone, Steven J. Collins, Diane Kofskey, Han Wang, Catriona A. McLean, Jean-Philippe Brandel, Nicolas Privat, Véronique Sazdovitch, Charles Duyckaerts, Tetsuyuki Kitamoto, Ermias D. Belay, Ryan A. Maddox, Fabrizio Tagliavini, Maurizio Pocchiari, Ellen Leschek, Brian S. Appleby, Jiri G. Safar, Lawrence B. Schonberger, Pierluigi Gambetti

**Affiliations:** 1 Departments of Pathology, Case Western Reserve University, School of Medicine, Cleveland, OH 44106 USA; 2 Departments of Neurology, Case Western Reserve University, School of Medicine, Cleveland, OH 44106 USA; 3 Departments of Psychiatry, Case Western Reserve University, School of Medicine, Cleveland, OH 44106 USA; 4 National Prion Disease Pathology Surveillance Center, Case Western Reserve University, School of Medicine, Cleveland, OH 44106 USA; 5 Inserm U1127, CNRS UMR 7225, Sorbonne Universités, UPMC Univ Paris VI UMR S 1127, Institut du Cerveau et de la Moelle épinière, Paris, France; 6 AP-HP, Cellule Nationale de Référence des maladies de Creutzfeldt-Jakob, Groupe Hospitalier Pitié-Salpêtrière, Paris, France; 7 AP-HP, Laboratoire de Neuropathologie R Escourolle, Groupe Hospitalier Pitié-Salpêtrière, Paris, France; 8 Department of Experimental, Diagnostic and Specialty Medicine, University of Bologna, Bologna, Italy; 9 IRCCS, Institute of Neurological Sciences, Bologna, Italy; 10 Fondazione IRCCS, Istituto Neurologico Carlo Besta, Milan, Italy; 11 Australian National Creutzfeldt-Jakob Disease Registry, Department of Medicine, and The Florey Institute of Neuroscience and Mental Health, The University of Melbourne, Parkville, 3010 Australia; 12 Department of Neurology, University Hospitals Cleveland Medical Center, Cleveland, OH 44106 USA; 13 Department of Anatomical Pathology, Alfred Health, Melbourne, 3181 Australia; 14 Victorian Brain Bank, the Florey institute of Neuroscience and Mental Health, The University of Melbourne, Parkville, 3010 Australia; 15 Department of Neurological Science, Tohoku University Graduate School of Medicine, Sendai, Japan; 16 Division of High-Consequence Pathogens and Pathology, National Center for Emerging and Zoonotic Infectious Diseases, Centers for Disease Control and Prevention, Atlanta, GA USA; 17 Department of Neurosciences, Istituto Superiore di Sanità, Rome, Italy; 18 National Institute of Diabetes and Digestive and Kidney Diseases, National Institutes of Health, Department of Health and Human Services, Bethesda, MD USA; 19 Department of Pathology, 4th floor, room 419, Case Western Reserve University, 2085 Adelbert Road, Cleveland, OH 44106 USA; 20 Department of Pathology, 4th floor, room 402C, Case Western Reserve University, 2085 Adelbert Road, Cleveland, OH 44106 USA

**Keywords:** Amyloid-β, Pathology, iCJD, Cerebral amyloid angiopathy, Thioflavin S

## Abstract

The presence of pathology related to the deposition of amyloid-β (Aβ) has been recently reported in iatrogenic Creutzfeldt-Jakob disease (iCJD) acquired from inoculation of growth hormone (GH) extracted from human cadaveric pituitary gland or use of cadaveric dura mater (DM) grafts.

To investigate this phenomenon further, a cohort of 27 iCJD cases – 21 with adequate number of histopathological sections – originating from Australia, France, Italy, and the Unites States, were examined by immunohistochemistry, amyloid staining, and Western blot analysis of the scrapie prion protein (PrP^Sc^), and compared with age-group matched cases of sporadic CJD (sCJD), Alzheimer disease (AD) or free of neurodegenerative diseases (non-ND).

Cases of iCJD and sCJD shared similar profiles of proteinase K-resistant PrP^Sc^ with the exception of iCJD harboring the “MMi” phenotype. Cerebral amyloid angiopathy (CAA), either associated with, or free of, Thioflavin S-positive amyloid core plaques (CP), was observed in 52% of 21 cases of iCJD, which comprised 37.5% and 61.5% of the cases of GH- and DM-iCJD, respectively. If only cases younger than 54 years were considered, Aβ pathology affected 41%, 2% and 0% of iCJD, sCJD and non-ND, respectively. Despite the patients’ younger age CAA was more severe in iCJD than sCJD, while Aβ diffuse plaques, in absence of Aβ CP, populated one third of sCJD. Aβ pathology was by far most severe in AD. Tau pathology was scanty in iCJD and sCJD.

In conclusion, (i) despite the divergences in the use of cadaveric GH and DM products, our cases combined with previous studies showed remarkably similar iCJD and Aβ phenotypes indicating that the occurrence of Aβ pathology in iCJD is a widespread phenomenon, (ii) CAA emerges as the hallmark of the Aβ phenotype in iCJD since it is observed in nearly 90% of all iCJD with Aβ pathology reported to date including ours, and it is shared by GH- and DM-iCJD, (iii) although the contributions to Aβ pathology of other factors, including GH deficiency, cannot be discounted, our findings increase the mounting evidence that this pathology is acquired by a mechanism resembling that of prion diseases.

**Electronic supplementary material:**

The online version of this article (10.1186/s40478-017-0503-z) contains supplementary material, which is available to authorized users.

## Introduction

The key pathogenic event of all prion diseases is the conversion of a normal or cellular prion protein (PrP^c^) into a misfolded and disease-associated isoform commonly identified as scrapie prion protein (PrP^Sc^) or prion. Newly converted PrP^Sc^ then propagates and accumulates preferentially in the central nervous system (CNS) typically accompanied by spongiform degeneration, gliosis and neuronal cell death. These pathogenetic features apply especially to Creutzfeldt-Jakob disease (CJD), by far the most common human prion disease. The pathogenic mechanism based on the conformational conversion of PrP^C^ into PrP^Sc^, which then acts as a seed, can conceptually accommodate not only PrP^Sc^ accumulation and propagation in the affected subject, but also the potential transmission of the process from affected to non-affected subjects. Although the sporadic and inherited forms account for the majority of human prion diseases [24], less than 1% of the prion diseases is acquired from animals or humans via an infectious mechanism [7]. Within this group, iatrogenic CJD (iCJD) is of particular interest. More than 492 cases of iCJD have been reported worldwide [4]. Most cases have been associated with the administration of growth hormone (GH) extracted from cadaveric pituitary glands and the application of cadaveric dura mater (DM) grafts [7]. The existence of iCJD and experimental evidence have firmly established the infectious property of prion diseases [18, 26, 27].

The central pathogenic event in Alzheimer’s disease (AD), the most common cause of dementia, is the deposition of amyloid β (Aβ), a truncated fragment of the amyloid precursor protein (*APP*), which leads to the formation of extracellular Aβ plaques [22, 30]. Deposition of highly phosphorylated, microtubule-associated tau protein (p-tau) also occurs [57] and, is believed to be a downstream event [33]. Most evidence indicates that deposition of Aβ and p-tau in the affected brain is stereotypical and hierarchical [5, 63]. More recently, it has been pointed out that Aβ and p-tau mimic several major characteristics of PrP^Sc^ including mechanisms of accumulation and propagation and formation of distinct Aβ and p-tau species that fulfill some of the characteristics of strains [16, 28, 56, 67, 68]. Like PrP^Sc^, Aβ has been detected in tissues other than CNS parenchyma including dura mater and pituitary glands [35, 43]. Furthermore, Aβ and p-tau pathologies (but not fully developed AD) have been replicated following Aβ or p-tau intracerebral or peripheral inoculations to transgenic mice that express human *APP* harboring AD pathogenic mutations as well as wild type human *APP*, indicating that Aβ pathology is transmissible [11, 20, 47, 48, 70]. In contrast to prion diseases, there is no evidence that AD and/or tauopathy (e.g., frontotemporal lobar degeneration) transmit from human-to-human.

Five recent studies have independently reported the presence of significant Aβ pathology in 4 of 8, 18 of 33, and 1 of 24 cases of iCJD from the United Kingdom (UK) and France, linked to injections of cadaveric GH (GH-iCJD) [19, 37, 53], and in 5 of 7 Swiss and Austrian iCJD and 13 of 16 Japanese iCJD cases who had received DM grafts (DM-iCJD) [23, 31]. In the most severely affected cases, Aβ amorphous aggregates were also detected in the pituitary gland and DM [37, 43]. The concomitant presence of Aβ and PrP^Sc^ deposits resulting in mixed AD and CJD phenotypes has been reported [8, 25, 50, 65]. However, while the AD-CJD mixed phenotype has been observed in older CJD-affected individuals [29, 64], most of the 28 iCJD cases harboring significant Aβ pathology were under 55 years of age [19, 23, 37, 53]. A possible explanation for the higher prevalence of Aβ pathology at a relatively young age in iCJD-affected subjects is that both Aβ and PrP^Sc^ seeds are transmitted during the iatrogenic procedures and then accumulate and propagate concurrently. However, Ritchie and coworkers have also observed brain Aβ pathology in absence of PrP^Sc^ deposition in recipients of cadaveric GH [53]. This important finding strongly argues for human-to-human transmission of an Aβ-related condition independently from the PrP^Sc^ seeding process. Nonetheless, whether the neuroendocrine deficiency may favor the Aβ pathology in some cases is still unknown [21].

To further investigate the iatrogenic seeding of Aβ pathology, we examined whether this condition occurs not only with UK and French GH as previously described but with receipt of GH produced before 1977 in the United States (US) as well. All US GH-iCJD patients to date received GH made before 1977, when a GH purification procedure was adopted that reduced or eliminated prion contamination. We also wanted to further examine the phenotypes of the DM-iCJD linked to the use of the Lyodura® brand. Out of 27 cases of definite iCJD, we selected 21 which were suitable for detailed histopathological examination and were provided by national prion surveillance centers of Australia, France, Italy, and the US. Cases of sporadic CJD (sCJD), non-neurodegenerative disorders (non-ND), and AD were used as comparison groups. Two autopsied cases who underwent GH treatment but did not develop iCJD were also examined. We report that (i) over 50% of iCJD cases harbored significant Aβ pathology, which included cerebral amyloid angiopathy (CAA) in all cases; (ii) the prevalence of the Aβ-positive iCJD subset was significantly higher than that of Aβ-positive sCJD cases, which were on average 17 years older; (iii) p-tau pathology was present but did not distinguish Aβ-positive iCJD cases from the sCJD controls, and seemed to be age-related; (iv) the phenotypic characteristics of the Aβ pathology in iCJD were distinct from those of typical AD.

## Materials and methods

### Reagents and antibodies

Dulbecco’s Phosphate Buffered Saline (DPBS), NaCl, Nonidet P-40, Sodium deoxycholate, Tris–HCl, Phenylmethanesulfonyl fluoride (PMSF), proteinase K (PK), Thioflavin S, and Kodak Biomax MR and XAR films were from Sigma-Aldrich (St. Louis, MO, USA). Tween 20, β–mercaptoethanol, Tris buffered saline (TBS), 2X Laemmli sample buffer, non-fat dry milk, 15% Criterion Tris–HCl polyacrylamide precast gels, 30% Acrylamide/Bis solution, tetramethylethylenediamine (TEMED), 10% sodium dodecyl sulfate (SDS) and ammonium persulphate (APS) were from Bio-Rad Laboratories (Hercules, CA, USA). Odyssey Blocking Buffer was from LI-COR Biosciences (Lincoln, NE, USA); polyvinylidene difluoride (PVDF) membrane (Immobilon-FL and Immobilon-P) was from EMD Millipore (Billerica, MA, USA). The primary antibodies (Abs) included the AT8 (to human phospho-tau residues Ser202 and Thr205) from Thermo Fisher Scientific Inc. (Waltham, MA, USA), 4G8 (to human Aβ residues 17–24), and 3F4 (to human PrP residues 106–110) [38, 71] from Richard Kascsak at the N.Y.S. Institute for Basic Research; 12B2 (to human PrP residues 89–93) [45] was from the Wageningen University & Research (Lelystad, Netherlands), whereas Tohoku-2 (to human PrP residues 97–103) [42] was kindly provided by Dr. Tetsuyuki Kitamoto. Secondary Abs were the sheep anti-mouse IgG from Life Sciences (Piscataway, NJ, USA), IRDye 800CW goat anti-mouse IgG (1 mg/ml) and IRDye 680RD goat anti-rabbit IgG (1 mg/ml) from LI-COR Biosciences (Lincoln, NE, USA). Reagents ECL and ECL plus were from GE Healthcare, Life Sciences (Piscataway, NJ, USA); Envision Flex Peroxidase Blocking Reagent, Envision Flex/HRP and Envision Flex DAB were from Dako (Dako North America Inc., Carpinteria, USA); the Vectashield mounting medium for fluorescence was from Vector Laboratories Inc. (Burlingame, CA, USA).

### Patients

Brain tissue from 27 confirmed iCJD patients was collected in this study. Thirteen of these cases were associated with cadaveric GH extracted from pituitary glands, whereas the other 14 cases were associated with cadaveric DM graft (Table 1 and Additional file 1: Table S1). All GH-iCJD were from the US and were treated with GH under the US National Hormone and Pituitary Program and started their treatment between 1969 and 1974. Tissue from 12 of the 13 US GH-iCJD was obtained at autopsy and one at biopsy. Two DM-iCJD were US cases, but only one -a 26 years old man -had received the Lyodura® brand dura (B. Braun Melsungen AG, Melsungen, Germany) (DM_L_-iCJD) whereas the other case -a 39 years old woman -had received the Tutoplast® brand dura (Pfrimmer-Viggo GmbH + Co, Erlangen, Germany) (DM_T_-iCJD). An important difference between these brands of dural graft is that unlike Tutoplast, Lyodura brand dural grafts were intermingled with many other dural grafts during the manufacturing procedure, thereby increasing the risk of cross-contamination. Other DM-iCJD cases included 1) two cases from the Australian National CJD Registry (Melbourne, Australia), 2) six cases from the Réseau National de Référence de maladies de Creutzfeldt-Jakob and Centre National de Référence des agents transmissibles non conventionnels (Paris, France), and 3) four cases from the Istituto Superiore di Sanita’ (Rome, Italy). With the exception of one French DM-iCJD case, for which the medical product Lyodura® brand could not be confirmed, all cases were DM_L_-iCJD. The histopathology and/or clinical and molecular features have been published for two US DM-iCJD (cases 1 and 2, Table 1) [3, 10, 32], three US GH-iCJD (cases 6, 9 and 10, Table 1) [10], and one Australian DM_L_-iCJD (case 17, Table 1) [58]. The five distinct comparison groups included 1) two US non-CJD patients who received the GH between years 1973–1977 (50 years) or between years 1977–1981 (46 years). The latter case may not have received any of the pre-1977 produced higher risk US GH material because 1977 was the transition year when a new purification procedure of GH extraction was started [1]; 2) 67 confirmed sCJD cases from Australia (*N* = 4), France (*N* = 11), Italy (*N* = 8) and US (*N* = 44) (Additional file 1: Tables S1 and S2); 3) 11 US autopsied cases of non-ND with age at death of 40 ± 12 years (range, 25–59 years) following diagnosis of blood cancer (*N* = 3; 25, 27 and 41 years), intracranial tumors (*N* = 2; 35 and 44 years), hemoglobin sickle cell disease (28 years), scrotal abscess and pulmonary embolus (31 years), type II diabetes mellitus (47 years), gastrointestinal hemorrhage (50 years), systemic lupus erythematosus (56 years), end stage renal disease and cardiac arrest (59 years); 4) seven US cases of AD with age at death of 64 ± 8 years, and disease duration of 75 ± 39 months [12]. Disease duration was not available in one AD case. All sporadic and iatrogenic CJD cases were classified according to Parchi and collaborators (1999). Since all Aβ-positive cases had multiple cortical sections not all of which were positive, we excluded from the study six Aβ-negative cases with only one section available.

**Table 1:** All examined cases of iCJD from countries of treatment with clinical, molecular and histopathological features

Case number	Iatrogenic exposure	Country	Sex	Age at death (years)	Disease duration (months)	Mean incubation period^a^ (years)	Codon 129 genotype	PrP^Sc^ type	Histopath. phenotype	Two or more histological sections
**1** ^b,c^	DM_L_	United States	M	26	5	19	na	na	MM(MV)1	yes
2^d,e^	DM_T_		F	39	4	6	MM	1	MM(MV)1	yes
3	GH		M	33	6	23	VV	na	VV2	yes
4	GH		M	39	17	24	MV	na	MV2K	yes
5	GH		M	39	7	27	MM	2	Atypical	yes
6^e^	GH		F	41	2	26	MM	1	MM(MV)1	yes
7	GH		M	43	26	23	MV	i+2	MV2K	yes
**8**	GH		M	44	18	32.5	MV	i+2	MV2K	yes
**9** ^e^	GH		M	51	14	38	MM	i	MMi	yes
**10** ^e^	GH		M	54	2	41.5	MM	1	MM(MV)1	yes
11	GH		M	23	19	10	na	na	MV2K or MMi	no
12	GH		M	34	18	23	na	na	MV2K or MMi	no
13	GH		M	37	3	21	na	na	MM(MV)1	no
14	GH		M	42	5	>28^f^	MM	na	Undetermined^g^	no
15^h^	GH		M	52	4	43	na	na	MM(MV)1	no
mean±SD^i^				41±8.5	11±8	28±9.5^j^				
**16**	DM_L_	Australia	M	32	3.5	16.5	MV	na	MM(MV)1	yes
**17** ^k^	DM_L_		M	62	2	5	na	na	MM(MV)1	yes
mean±SD				47±21	3±1	11±8				
18	DM_L_	France	M	25	8	8	MV	na	MM(MV)1	yes
**19**	DM_Unk_		M	29	6	25	MV	1	MM(MV)1	yes
20	DM_L_		M	42	5	6	VV	na	VV2	yes
**21**	DM_L_		F	50	14	11	MM	na	MMi	yes
**22**	DM_L_		F	62	4	4	VV	na	VV2	yes
**23**	DM_L_		F	71	4	4	MV	na	MV2K	yes
mean±SD				46.5±18	7±4	10±8				
24	DM_L_	Italy	F	23	27	21	VV	2	VV2	yes
25	DM_L_		F	26	6	12	MM	na	MM(MV)1	no
26	DM_L_		M	42	3.5	18	MM	1+2	MM1+2	yes
**27**	DM_L_		M	75	3	18	VV	2	VV2	yes
mean±SD				41.5±24	10±11.5	17±4				

^a^The mean incubation period was measured from the mid-point of GH therapy or date of receipt of the dura graft to the clinical onset of the disease; ^b^[3]; ^c^Numbers in bold indicate iCJD cases with Aβ-positive pathology; ^d^[32]; ^e^ [10]; ^f^Unknown starting date for GH treatment (incubation period thought to be greater than 28 years); ^g^Associated with severe spongiosis and gliosis; ^h^Biopsy; ^i^Mean±SD of the US iCJD cases does not include DM_L_ (case 1) and DM_T_ (case 2); ^j^It does not include case 14; ^k^[58]; Histopath. histopathological, *DM*_Unk_ dura mater of an unknown (Unk) brand, *na* not available *SD* standard deviation

All of the US iCJD and the two CJD-free recipients of GH were collected at the National Prion Disease Pathology Surveillance Center (NPDPSC) in Cleveland (OH) in collaboration with the National Institute of Diabetes, Digestive and Kidney Diseases, NIH, (Bethesda, MD) and Division of High Consequence Pathogens and Pathology, CDC, (Atlanta, GA) and Westat Agency (Rockville, MD). The AD and US sCJD controls were from the NPDPSC whereas the 11 non-ND cases were collected in the repository of the Department of Pathology at Case Western Reserve University. The neuropathological study was carried out either in our laboratory or in the laboratories of the participating countries. Neuropathology was reviewed by IC and MC. Western blot (WB) examination of iCJD cases was performed in our laboratory in Cleveland (cases 2, 6–10, 24, 26 and 27, Table 1), at University of California, San Francisco (UCSF) (case 5) as well as in France (case 19, Table 1) and Italy (cases 24, 26 and 27, Table 1).

### Histology and immunohistochemistry

Formalin-fixed brain tissue was treated as previously described [9]. Briefly, sections were deparaffinized and rehydrated, immersed in 1X Tris buffered saline-Tween 20 (TBS-T), and endogenous peroxidase blocked after incubation with the Envision Flex Peroxidase Blocking Reagent for 10 minutes (min). Sections were washed, immersed in 1.5 mmol/L hydrochloric acid, microwaved for 15 min and probed with Abs 3F4 (1:1000), 4G8 (1:3000), and AT8 (1:200) for 1 hour (h). After washing and incubation with Envision Flex/HRP polymer for 30 min, sections were treated with Envision Flex DAB to show the immunostaining.

### Thioflavin S staining

After deparaffinization, formalin-fixed sections were stained in Thioflavin S for 7 min, washed three times in 80% alcohol, dehydrated in ethanol, cleared in xylene, and cover slipped with Vectashield mounting medium for fluorescence. Sections were kept in the dark at 4 °C for 30 min before being viewed under the fluorescence microscope (Olympus IX71).

### Evaluation of the histopathological changes associated with Alzheimer’s disease and Prion disease

Histopathological evaluation was performed in 10 or more anatomical regions in most cases. Standard brain locations included the frontal, temporal, parietal, occipital and entorhinal cortices, hippocampus, striatum, thalamus, midbrain and cerebellar hemispheres and/or vermis. Histopathological evaluation included 1) Hematoxylin-eosin (HE) staining, to assess the presence of spongiform degeneration, gliosis, and amyloid Aβ cores; 2) Immunostaining with Abs 4G8, AT8 and 3F4 to Aβ, p-tau and PrP, respectively; 3) Staging of Aβ plaques using monoclonal Ab 4G8 and Thioflavin S, according to Thal et al. [63]. This method identifies five major stages or phases of Aβ plaques deposition affecting the neocortex, including frontal, temporal, parietal and occipital cortices (Phase 1), hippocampus and entorhinal cortex (Phase 2), striatum thalamus and midbrain (Phase 3–4), and cerebellum (Phase 5); 4) Description of the morphology of the Aβ plaques, including a) diffuse plaques, b) core plaques (CP) (i.e., a plaque with a dense core surrounded by a halo and a corona of lightly stained Aβ), c) neuritc plaques (i.e., a core plaque surrounded by p-tau dystrophic neurites); 5) Staging of Aβ CAA, according to previous procedures [61] with recognition of three major phases: CAA affecting the neocortex (Phase 1), hippocampus, entorhinal cortex, cerebellum and midbrain (Phase 2), striatum and thalamus (Phase 3); 6) Typing of CAA, identified as CAA type 1 or type 2 depending on the presence or absence of Aβ deposits in the cortical capillaries [60]. The criteria for the identification of CAA type 1 were i) diameter of the vessels ≤ 10 μm, and ii) deposition of Aβ in the outer basement membrane; 7) Severity of CAA, based on a modified protocol by Vonsattel and coworkers that uses 4G8-stained sections (with the addition of Thioflavin S in some cases) instead of Congo red [66]; 8) Brain distribution of neurofibrillary tangles (NFT) and neocortical distribution of dystrophic neurites (DN); 9) Severity of NFT and DN expressed as NFT or DN density in one microscopic field (area: 1.3 × 1.0 mm^2^, using a 10X objective) harboring the highest density of NFT or DNs in one or more brain regions; severity was scored as mild (≤ 10 NFT or DN), moderate (> 10 to < 30 NFT or DN) and severe (≥ 30 NFT or DN); 10) Automated image acquisition and morphometric analysis to assess density (expressed as the percentage of the area of the cerebral cortex occupied by plaques) and size (diameter) of Thioflavin S-positive Aβ core plaques as well as size (perimeter) of the blood vessel; 11) Semiquantitative analysis of the percentage of 4G8-positive Aβ deposits along the wall of blood vessels; and 12) Double immunostaining with 4G8 and 3F4 to rule out the co-localization of PrP and Aβ [25].

### Image acquisition and statistical analysis

Image acquisition was carried out with a Leica DFC 425 digital camera mounted on a Leica DM 2000 microscope. Images were analyzed by the software Image-Pro Plus 7.0 (Media Cybernetics, Inc.). Cumulative survival curves were generated by the Kaplan–Meier analysis. Statistical significance between the survival curves of the individual groups were determined by the log rank (Mantel-Cox) test. When comparing different patient groups, *P*-values were calculated with Chi-square test, Fisher’s exact test, Student’s t-test (two-tailed). All the statistical analyses were performed using GraphPad Prism 6.0.

### Preparation of brain homogenates, proteinase K digestion and Western blot analysis

10% (wt/vol) brain homogenates (BH) prepared in 1X LB100 buffer (100 mM NaCl, 0.5% Nonidet P-40, 0.5% sodium deoxycholate, 10 mM EDTA, 100 mM Tris–HCl, pH 6.9 at 37 °C), were centrifuged at 1000 x g for 5 min at 4 °C, pellets discarded and supernatants (S1) collected. S1 aliquots were incubated with 100 U/ml PK at 37 °C for 1 h [PK specific activity was 48 U/mg at 37 °C, with 1 U/ml equal to 20.8 μg/ml PK]. The enzymatic digestion was stopped with PMSF (3 mM final concentration). Each sample was diluted with an equal volume of 2X Laemmli sample buffer (6% SDS, 20% glycerol, 4 mM EDTA, 5% β –mercaptoethanol, 125 mM Tris–HCl, pH 6.8) and denatured at 100 °C for 10 min. Proteins were separated on 15% Criterion™ Tris–HCl Precast Gels (W x L: 13.3 cm × 8.7 cm) at 120 Volts (V) for 20 min followed by 150 V for 1 h 45 min, or using 15% Tris–HCl SDS–polyacrylamide gels (W x L: 20 cm × 20 cm) at 25 mA/gel for 1 h 45 min followed by 35 mA/gel for 6 h 30 min (Bio-Rad PROTEAN® II xi cell system). For near-infrared WB analysis, proteins were blotted onto the Immobilon-FL PVDF membrane for 2 h, blocked with the Blocking Buffer Odyssey for 45 min and incubated with Abs 3F4 (1:20,000), 12B2 (200 ng/ml) or Tohoku-2 (1:10,000) for 2 h. Membranes were then washed with 1X DPBS containing 0.1% of Tween 20 (1X DPBS-T) and incubated with Abs IRDye 800CW goat anti-mouse IgG (1:15,000) or IRDye 680RD goat anti-rabbit IgG (1:15,000) for 1 h. After washing in 1X DPBS-T, membranes were developed with the Odyssey infrared imaging system (LI-COR Biosciences) as described by the manufacturer. For chemiluminescence, proteins were blotted onto the Immobilon-P PVDF membrane, blocked with 5% non-fat dry milk in 0.1% Tween 20-1X TBS (blocking buffer), and incubated with primary Abs and horseradish peroxidase-conjugated goat anti-mouse secondary Ab (1:3000). Membranes were developed by the enhanced chemiluminesce reaction using ECL and ECL plus reagents, and signal captured on MR and XAR films.

### Clinical evaluation

Medical records were reviewed by a clinician (BSA) and data were collected on demographics (age at death, gender, and race/ethnicity). Disease onset was defined as the time at which the first persistent and consistent symptom of prion disease was observed. Data on family history of dementia as well as past medical and surgical history were also collected. The mean incubation period in iCJD was measured from the mid-point of GH therapy or date of receipt of the DM graft to the clinical onset of the disease.

### Genetic analysis

DNA was extracted from frozen brain tissues and *APP*, presenilin 1 (*PSEN1*), presenilin 2 (*PSEN2*), and *PRNP* gene analysis was performed as previously described using Illumina and Sanger Sequencing for exons 4 and 5 in *PSEN1* [12]. Sequencing analysis was performed using Mutation Surveyor Version 4.0.7 (Softgenetics, State College, PA). Genotyping of Apolipoprotein E (*ApoE*) single-nucleotide polymorphisms was performed by Sanger sequencing (Center for Human Genetics, Cleveland, OH).

## Results

### Demographics, molecular features and histopathological phenotype of iCJD and controls including sCJD

We examined 27 cases of iCJD linked to cadaveric DM graft (*N* = 14) or GH (*N* = 13), who received the iatrogenic treatment in Australia, France, Italy and the US (Table 1). Sixty-seven cases of sCJD obtained from the same countries as the iCJD cases were used as controls (Additional file 1: Table S2). The sCJD case population was selected to be as similar to the age of the iCJD case population as possible. As expected, the iCJD cases had a higher percentage of premorbid neurological conditions (including intracranial tumors and head trauma) and neurosurgery given that these conditions often led to treatment with GH and/or DM grafts. No statistical correlations were found when intracranial tumor or head trauma were tested against either Aβ or tau pathologies in the iCJD and sCJD populations (Additional file 1: Table S3). Both iCJD and sCJD cohorts were compared to a seven case group with typical AD (Tables 2 and 3, and Additional file 2: Table S6). The iCJD cases were stratified by country of origin, age at death, disease durations and incubation period (Table 1). The data stratified according to diagnosis, i.e. all-iCJD, GH-iCJD, DM-iCJD and sCJD, as well as according to the age at death, i.e. ≤ 54 years (“young”) and > 54 years (“old”) are shown in Additional file 1: Table S1 and Additional file 3: Figure S1. For all the cases combined, the three iCJD diagnostic groups revealed no significant difference in mean age at death, disease duration and median survival, while the incubation period was significantly shorter in DM-iCJD than in GH-iCJD (*P* < 0.0001) (Additional file 1: Table S1 and Additional file 3: Figure S1). Separation of cases into young and old subsets underscored the younger age of the GH-iCJD cases, which exclusively populated the young group, and the wide age range of DM-iCJD (Additional file 1: Table S1). In the young group, the incubation period again was significantly shorter in DM-iCJD than GH-iCJD (*P* < 0.002). Overall, disease duration and incubation period were significantly shorter in the older group (*P* < 0.0008 and *P* < 0.03, respectively) (Additional file 1: Table S1). The sCJD control population was 8 years older at death than the iCJD population while disease duration was comparable in the two conditions. Sporadic CJD young cases had a mean age at death comparable to that of GH-iCJD while disease duration in old cases matched that of DM-iCJD (Additional file 1: Table S1).

**Table 2:** Staging and amyloid presence in Aβ CP from iCJD associated with Aβ pathology along with cases of sCJD and AD used as controls

Case number	Age (y)	Disease durat. (mo)	Codon 129 genotype	PrP^Sc^ type	Phase 1		Phase 2		Phase 3-4		Phase 5	
					Neocortex	Thiofl	Hippoc.	Thiofl	Subcortical	Thiofl	Cerebellum	Thiofl
iCJD												
1 (1)^a^	26	5	na	na	+++^b,c^	+^d^	++	-	+^e^	-	+	-
2 (19)	29	6	MV	1	+++^f^	+	+	+	-	nt	-	nt
3 (8)	44	18	MV	i+2	++++	+	++	-	+++	-	-	-
4 (9)	51	14	MM	i	-	-	-	-	-	nt	+	+
5 (17)	62	2	na	na	++++	+^g^	na	na	na	na	-	-
mean±SD	42±15	9±7			78%^h^		62.5%		40%		40%	
sCJD												
1 (57)^i^	63	3	MV	1	+++^f^	+	++	+	++	nt	-	nt
2 (59)	71	9	MV	2	+^f^	+	-	-	-	nt	-	nt
3 (65)	71	10	VV	2	+	+	-	nt	-	nt	-	nt
4 (67)	79	6	VV	2	+++	+	-	nt	-	nt	-	nt
mean±SD	71±6.5	7±3			57%		25%		17%		0%	
*P* value^j^					NS		NS		NS		NS	
AD												
1	56	60	na	na	++++	+	++	+	+++	+	+	+
2	56	60	na	na	++++	+	++	+	+++	+	+	+
3	59	na	na	na	++++	+	++	+	+++	+	+	+
4	60	130	na	na	++++	+	++	+	+++	+	+	-
5	70	66	na	na	++++	+	++	+	+++	+	+	+
6	72	111	na	na	++++	+	++	+	+++	-	+	+
7	74	22	na	na	++++	+	++	+	+++	+	+	+
mean±SD	64±8	75±39			100%		100%		100%		100%	
*P* value^k^					< 0.02		< 0.04		< 0.0004		< 0.05	

Phases 1 to 5 refer to Aβ plaques deposition in various brain regions reflecting disease progression according to Thal et al [63]; ^a,i^Numbers in parenthesis in the first column refer to numerals used in ^a^Table 1 and ^i^Additional file 1: Table S2; ^b^Minus and plus signs indicate absence (-) or presence (+) of Aβ pathology affecting one (+), two (++), three (+++) or four (++++) of the brain regions that characterize each phase; Phase 1: frontal, temporal, parietal and occipital cortical regions; Phase 2: hippocampus and entorhinal cortex; Phase 3-4: striatum, thalamus and midbrain; Phase 5: cerebellum; ^c,e^Refers to one iCJD with one or two missing sections from the ^c^neocortex or ^e^subcortical regions; ^d^The (+) sign under Thiofl (Thioflavin S) indicates presence of amyloid plaques in at least one brain region; the (-) sign indicates that all regions tested had Thioflavin S negative staining; ^f^Refers to one iCJD and two sCJD cases with missing parietal cortex; ^g^Positive staining with Congo red [58]; ^h^Percentage of brain regions with CP; ^j,k^Fisher’s exact test comparing iCJD to ^j^sCJD or ^k^AD; *Hippoc.* hippocampal formation (hippocampus and entorhinal cortex), *y* years, *durat.* duration, *mo* months, *na* not available, *nt* not tested, *NS* not significant, *SD* standard deviation

**Table 3:** Staging of Aβ CAA in iCJD and control cases of sCJD and AD

Case number	Age (y)	Disease duration (mo)	Codon 129 genotype	PrP^Sc^ type	Phase 1		Phase 2					Phase 3		Vstl.^a^ score	CAA^b^ type
					Neocortex	Thiofl	Hippoc.	Thiofl	Crbl	Thiofl	Midb.	Subcort.	Thiofl		
iCJD															
1 (1)^c^	26	5	na	na	+++^d,e^	+^f^	++	+	+	+	na	-^e^	-	3	1
2 (19)	29	6	MV	1	+++^g^	+	+	+	+	nt	+	+	nt	2	2
3 (16)	32	3.5	MV	na	+^h^	na	-	na	-	na	-	-	na	1	2
4 (8)	44	18	MV	i+2	++++	+	++	-	+	+	-	-	-	3	2
5 (21)	50	14	MM	na	+++^g^	+	-	nt	-	nt	+	+	nt	1	2
6 (9)	51	14	MM	i	++	+	-	-	+	+	-	-	-	2	2
7 (10)	54	2	MM	1	+	+	-	-	-	-	-	-	nt	1	2
8 (17)	62	2	na	na	++++	+^i^	na	na	+	+^i^	na	na	na	na	na
9 (22)	62	4	VV	na	++^g^	-	-	nt	+	-	+	+	nt	1	2
10 (23)	71	4	MV	na	-	nt	+	-	+	-	-	-	nt	1	2
11 (27)	75	3	VV	2	++++	-	+	-	+	-	-	-	nt	1	2
mean±SD	50.5±17	7±6			69%^j^		35%		73%		33%	16%			
sCJD															
1 (55)^k^	49	4	MM	1	+^g^	-	-	-	-	nt	-	-	nt	1	2
2 (56)	63	4	MM	1	+^g^	-	++	-	+	-	+	++	nt	1	2
3 (57)	63	3	MV	1	-	-	-	-	+	nt	-	+	nt	1	2
4 (44)	64	1	MM	1	++	+	-	-	-	nt	-	-	-	1	2
5 (58)	71	8	VV	2	+++^g^	-	++	-	+	-	-	-	nt	1	2
6 (66)	74	4.5	VV	2	+	-	-	nt	-	nt	-	-	nt	1	2
mean±SD	64±9	4±2			38%		33%		50%		17%	25%			
*P* value^l^					< 0.03		NS		NS		NS	NS		= 0.05*	NS*
AD															
1	56	60	na	na	++++	+	-	-	+	+	+	+	-	2	2
2	56	60	na	na	++++	+	-	-	+	+	-	-	-	3	2
3	59	na	na	na	++++	-	-	-	+	+	-	-	-	3	2
4	60	130	na	na	++++	-	-^n^	-	+	-	-	+	+	2	2
5	70	66	na	na	++++	+	+	-	+	+	-	-	-	2	2
6	72	111	na	na	++++	+	+	+	+	+	+	+	+	3	1
7	74	22	na	na	+++	-	-	-	+	+	-	+	-	3	2
mean±SD	64±8	75±39			96%		15%		100%		29%	29%			
*P* value^m^					< 0.006		NS		NS		NS	NS		< 0.02*	NS*

Phases 1 to 3 refer to Aβ CAA deposition in various brain regions, reflecting disease progression according to Thal et al [61]; ^a^Vonsattel (Vstl) 0-3 score of Aβ CAA severity to the highest score in one or more brain locations [66]; ^b^Presence (type 1) or absence (type 2) of Aβ deposits in the cortical capillaries [60]; ^c,k^Numbers in parenthesis in the first column refer to numerals used in ^c^Table 1 and ^k^Additional file 1: Table S2; ^d^Minus and plus signs indicate absence (-) or presence (+) of Aβ CAA affecting one (+), two (++), three (+++) or four (++++) brain regions in each phase; Phase 1: frontal, temporal, parietal and occipital cortical regions; Phase 2: hippocampus, entorhinal cortex, cerebellum and midbrain; Phase 3: striatum, thalamus; ^e^Refers to one iCJD patient with one missing section from neocortex and subcortical regions; ^f^The (+) sign under Thiofl (Thioflavin S) indicates presence of positive staining of at least one brain region; the (-) sign indicates that all regions examined had Thioflavin S negative staining; ^g^Refers to 3 iCJD and 3 sCJD cases with missing parietal cortex; ^h^Refers to one iCJD with missing frontal cortex; ^i^Positive staining with Congo red [58]; ^j^Percentage of brain regions with Aβ CAA; ^l,m^Fisher’s exact test comparing iCJD to ^l^sCJD or ^m^AD; ^n^Refers to one AD patient with missing entorhinal cortex; *y* years, *mo* months *Hippoc.* hippocampal formation (hippocampus and entorhinal cortex), *Crbl* cerebellum, *Midb.* midbrain, *Subcort.* subcortical regions (striatum and thalamus), *na* not available, *nt* not tested, *NS* not significant, *SD* standard deviation; *Student’s t-test

**Fig. 1:**
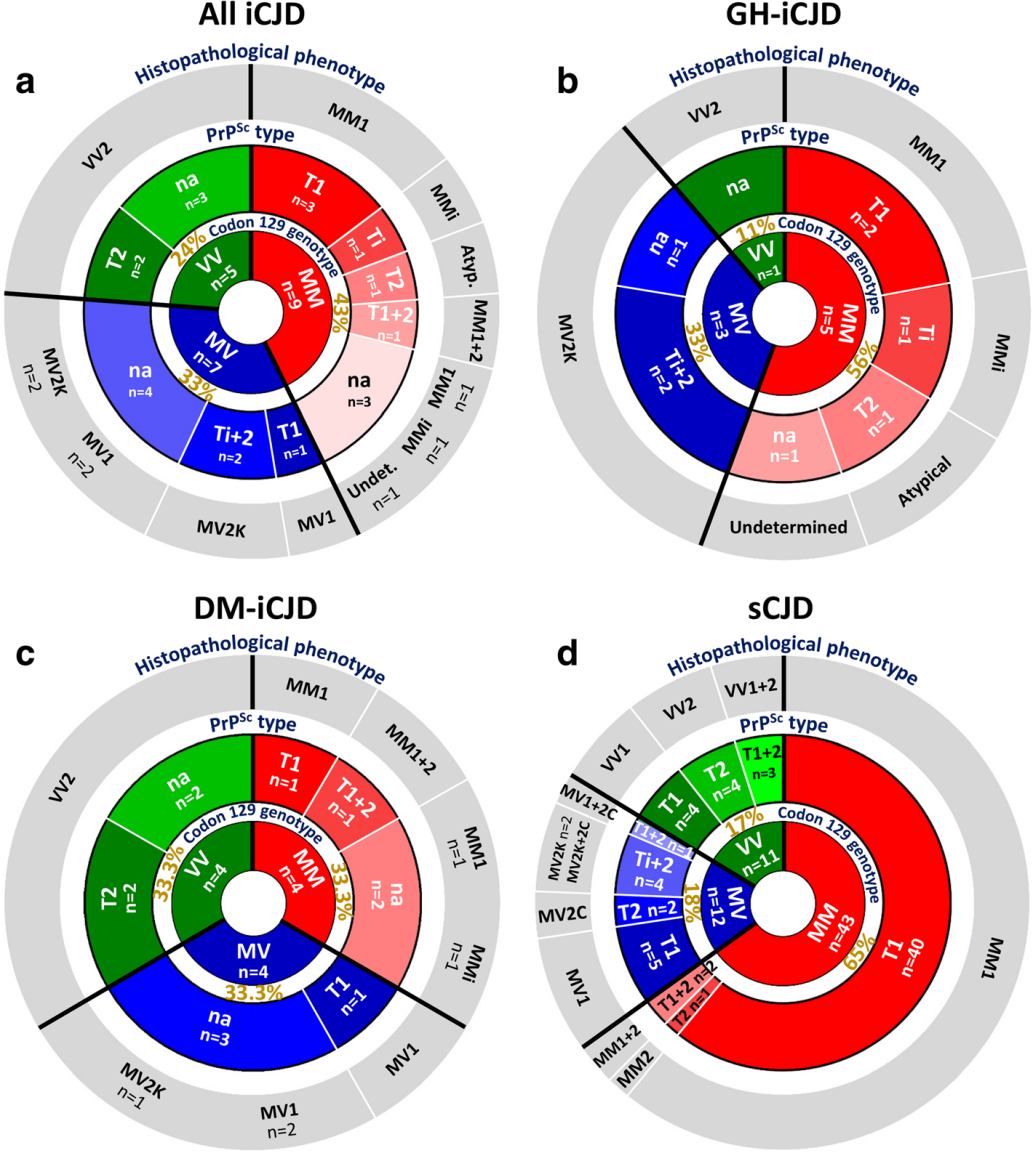
Ring doughnut charts visualizing distributions of codon 129 genotypes, resPrP^Sc^ types and disease phenotypes among iCJD and sCJD cases examined. **a** whole iCJD cohort; inner circle: percent distribution of codon 129 genotype (MM, MV, VV); intermediate circle: distribution of resPrP^Sc^ types (T1, T2, Ti, Ti ± 2, T1 + 2); outer circle: distribution of histopathological phenotype. **b** GH-iCJD, legend as **a**; **c** DM-iCJD, legend as **a**; **d** sCJD. T1: type 1; Ti: type intermediate; T2: type 2; Ti + 2: type i + 2; T1 + 2: type 1 + 2; n: number of cases; na: not available; Atyp.: atypical; Undet.: undetermined. Percentages refer to the distribution of codon 129 genotype

With regard to the molecular features, the percent distribution of the three genotypes (MM, MV, VV) at codon 129 of the PrP gene in the iCJD population (MM 43%, MV 33%, VV 24%) significantly differed from that of the sCJD controls (MM 65%, MV 18%, VV 17%) as well as those reported for unselected sCJD populations (MM 71%, MV 12%, VV 17%) [15] pointing to an increased relative prevalence of the MV and VV genotypes at the expense of the MM genotype in our iCJD cohort (Fig. 1). The iCJD genotype distribution also differed from those of Western European, Australian and the North American general populations (MM 37%, MV 51%, VV 12%) [14]. Keeping in mind the limited number of cases for some of these analyses, 129 genotype distribution also diverged in the GH-iCJD and DM-iCJD subsets: while the relative representation of the MV genotype was increased in both subsets, the DM-iCJD showed a three times higher representation of the VV genotype (33% vs. 11%) (Fig. 1). Analyses of the PK-resistant PrP^Sc^ (resPrP^Sc^) type distribution showed that in iCJD the combined representation of types 2 (with unglycosylated resPrP^Sc^ of ~19 kDa), “intermediate” (i; ~20 kDa), i + 2 (~20 + 19 kDa), and 1 + 2 (~21 + 19 kDa) (63%) was much higher than the representation of combined type 2 and i + 2 (28%) in sCJD unselected populations [15, 49], as well as in our sCJD controls, a finding consistent with the combined prevalence of the MV and VV genotypes which often are paired with type 2 and i + 2 (Fig. 1). Next, we compared phenotypic characteristics, such as disease duration and histopathological features in age-group matched cases of iCJD and sCJD controls which shared both 129 genotype and PrP^Sc^ type. Iatrogenic CJD with genotypes 129MM coupled with PrP^Sc^ type 1 (iCJDMM1), the only subset with a significant number of age-group matched cases, showed shorter mean disease duration than sCJDMM1 (2.7 vs. 5.6 months; *P* > 0.05). As for the histopathological features, when histopathological phenotypes associated with the same pairing of the 129 genotype and resPrP^Sc^ type were compared, all iCJD phenotypes were found to match the phenotypes of the corresponding sCJD, except for three cases. Two of these cases (GH-iCJD case 9 and DM-iCJD case 21), both harboring the 129MM genotype, had a phenotype characterized by the presence of kuru plaques resembling sCJDMV2K phenotype, which is typically not seen in sCJDMM associated either type 1 or 2 (Table 1). However, both these cases matched the previously described iCJDMMi phenotype [10, 39–41, 54]. The phenotype of the third case (GH-iCJD, case 5), which was associated with the 129MM genotype and resPrP^Sc^ type 2, differed in several features from typical phenotype of sCJDMM2. While spongiform degeneration type mimicked that of sCJDMM2, it was limited to the first cortical layers, rather than being widespread in the cerebral cortex, and it was accompanied by severe neuronal loss and astrogliosis while kuru plaques were lacking; furthermore, PrP immunostaining was punctate rather than forming coarse granular deposits (Additional file 4: Figure S2).

### Western blot profiles of the resPrP^Sc^ in iCJD and matching sCJD controls

**Fig. 2:**
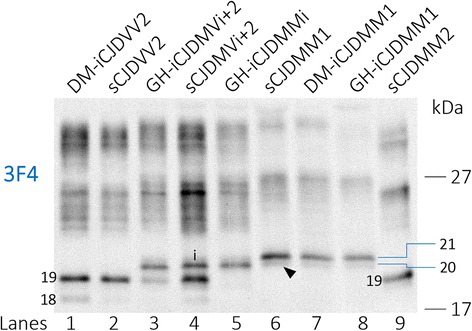
WB profile of resPrP^Sc^ from iCJD and sCJD controls using high resolution gel electrophoresis. BHs from the frontal cortex (lanes 8 and 9) and cerebellum (lanes 1–7) treated with 100 U/ml PK (~2000 μg/ml) were probed with 3F4. PrP bands were resolved in a 15% Tris–HCl, 20 cm-long gel, and visualized with the near-infrared LI-COR system. The resPrP^Sc^ profiles from iCJD (lanes 1, 3, 5, 7 and 8) and matching sCJD subtypes (lanes 2, 4 and 6) are similar as shown by the co-migration of the unglycosylated resPrP^Sc^ type 2 (19) (lanes 1–4) and type 1 (21) (lanes 6–8). The different thickness of the type 2 (19) band is within the variability range for this subtype. The unglycosylated resPrP^Sc^ band of ~20 kDa, also identified as type i (i), is visible in GH-iCJDMVi + 2 (lane 3), sCJDMVi + 2 (lane 4) and GH-iCJDMMi case (lane 5); a thin band of ~20 kDa (arrowhead) is also present in CJDMM1. An ~18 kDa fragment (18) is also present in CJDVV2 and CJDMVi + 2

The profiles of resPrP^Sc^ from 10 iCJD suitable cases (2, 6–10, 19, 24, 26 and 27, Table 1) were compared with those of sCJD cases harboring the same 129 genotype and resPrP^Sc^ type (Fig. 2). This comparative study revealed that resPrP^Sc^ profiles from nine of the 10 iCJD cases matched those of the corresponding sCJD subtypes (Fig. 2). Thus, probing with 3F4, all CJDVV2 cases showed an unglycosylated resPrP^Sc^ fragment of ~19 kDa, which typically identifies type 2, whereas CJDMVi + 2 cases showed the type i ~20 kDa fragment in addition to the unglycosylated resPrP^Sc^ of ~19 kDa. In both iCJDMM1 and sCJDMM1, resPrP^Sc^ was characterized by the type 1 (~21 kDa) fragment as well as a faint band of ~20 kDa (Fig. 2 and Additional file 5: Figure S3). Conversely, iCJDMMi (case 9, Table 1) showed a prominent fragment matching type i and a faint band of ~21 kDa possibly representing type 1 (Fig. 2 and Additional file 5: Figure S3). These findings confirm the presence of type i in iCJDMMi and CJDMVi + 2 [39, 54], and indicate that in small amounts, a ~20 kDa fragment matching type i in mobility may be present also in iCJDMM1 and sCJDMM1 while type 1 may be present in iCJDMMi (Fig. 2 and Additional file 5: Figure S3). Tissue was not available for a detailed profile determination of resPrP^Sc^ from iCJDMM case 5 (Table 1), but it was reported to be matching resPrP^Sc^ type 2.

### Aβ in iCJD, sCJD, prion disease free GH recipients and non-ND: Prevalence, clinical molecular and phenotype features

**Fig. 3:**
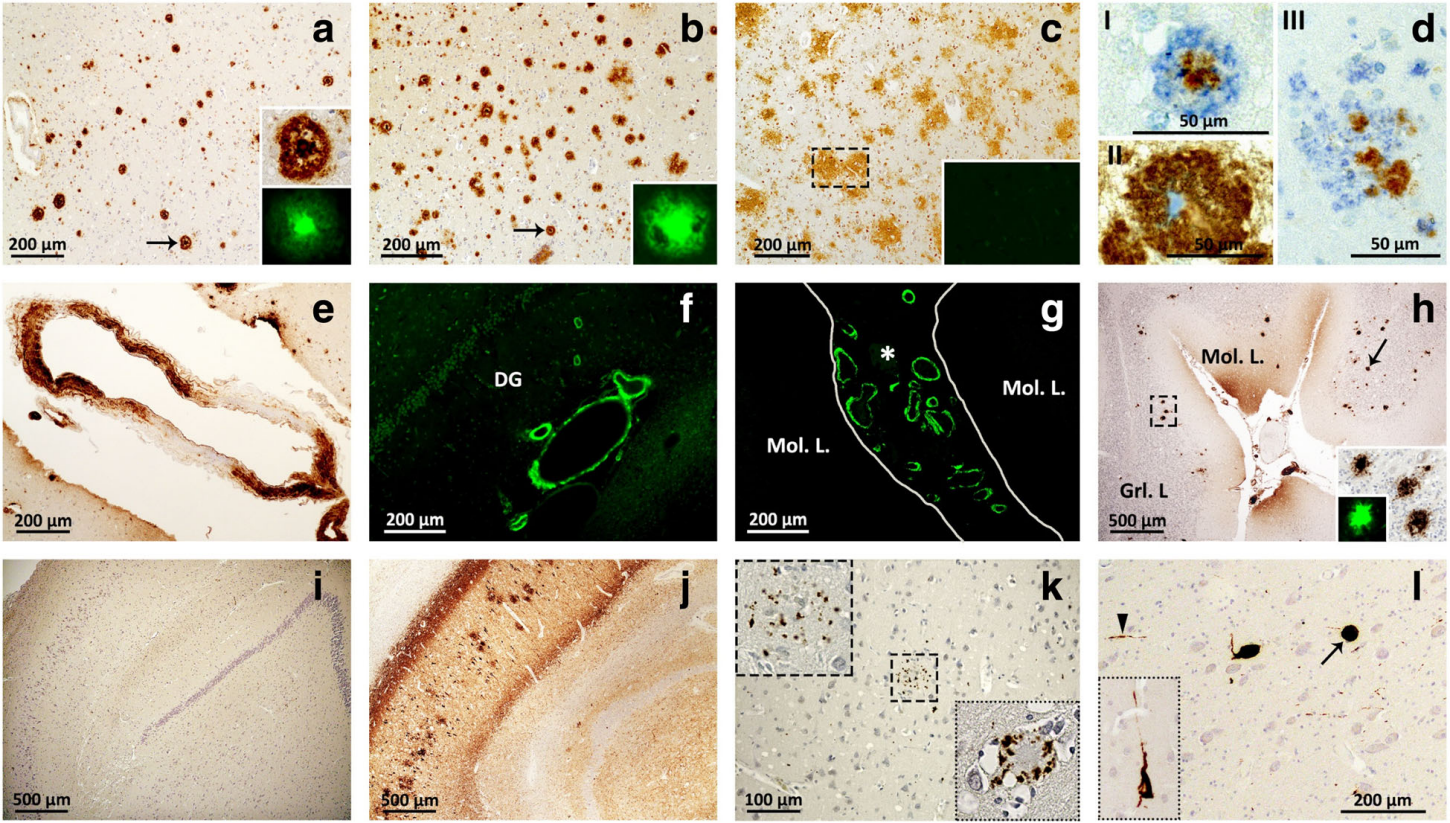
Aβ and tau pathology of iCJD, sCJD and AD. **a** and **b** typical Aβ CP (arrows) from the frontal cortex of iCJD (**a**) and AD (**b**); inset **a**: Aβ CP at higher magnification immunoprobed for Aβ (top) or stained with Thioflavin S (bottom); inset **b**: Thioflavin S-positive Aβ CP. **c** diffuse plaques from the frontal cortex of a case of sCJD; inset: Thioflavin S-negative staining of plaques (dashed rectangle). **d** co-localization of Aβ (cyan dye) and PrP (brown dye) immunoreactivity in the same plaque from cerebral (I and III) and cerebellar (II) cortices of iCJD. I: plaque exhibiting a PrP-positive core surrounded by an Aβ immunoreactive crown; II-III: plaques showing either the immunostaining opposite to that of I, with Aβ-positive core and a PrP immunoreactive crown (II), or random distribution of PrP- and Aβ-reactive aggregates (III). **e**-**g** Aβ deposits (**e**) and Thioflavin S-positive staining (**f** and **g**) of vessels in frontal cortex (**e**), hippocampus (**f**) and cerebellum (**g**) in iCJD; **f**: DG, Dentate gyrus; **g**: asterisk, Aβ CAA in the subarachnoid space between two cerebellar folia. **h** Aβ CP predominantly affecting the granular layer (Grl. L) and Purkinje layer of the cerebellum in iCJD (case 9, Table 1); arrow indicates Aβ CP; large and small insets: enlargements of Aβ CP identified by the dashed rectangle in the main figure and a Thioflavin S-positive Aβ CP, respectively. **i**-**j** hippocampus free of tau pathology in iCJD (**i**) but severely affected in AD (**j**). **k** Tau-positive DN associated with an Aβ CP in frontal cortex of iCJD (case 8, Table 1) (dashed square); top inset: enlargement of tau reactive DN identified in dashed square; bottom inset: DN associated with a PrP kuru plaque in iCJD (occipital cortex, case 9, Table 1). **l** globose NFT (arrow) and neuropil threads (arrowhead) in the nucleus basalis of Meynert of iCJD (case 6, Table 1); inset: flame-shaped NFT in the temporal neocortex of the same iCJD case. Abs: 3F4 (**d**), 4G8 (**a**-**e**, **h**) and AT8 (**i**-**l**) to PrP, Aβ and phosphorylated tau, respectively; Thioflavin S to Aβ (**a**-**c**, **f**-**h**)

**Fig. 4:**
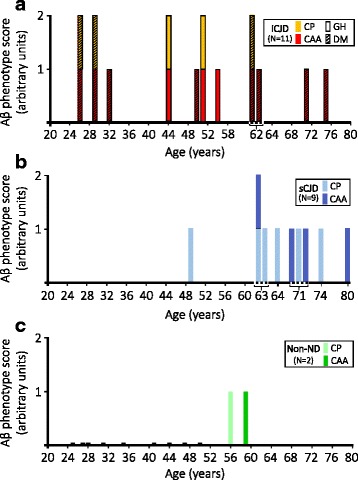
Aβ phenotype and age distribution in Aβ-positive iCJD and control cases. **a**: iCJD (*N* = 11); **b**: sCJD (*N* = 9); **c**: non-ND (*N* = 2). Scores for the Aβ phenotype is relative to the presence of either Aβ CP or CAA (score 1), or combinations of both (score 2). Brackets below dotted line in the X-axis underline cases of the same age; GH: growth hormone; DM: dura mater

**Fig. 5:**
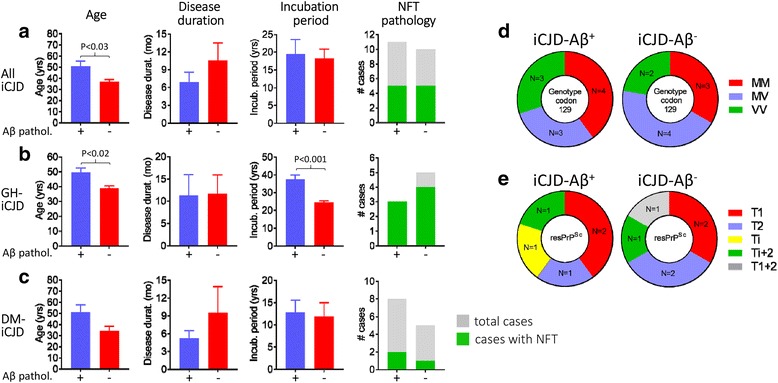
Graphic representation of demographic data, NFT pathology, codon 129 genotype and resPrP^Sc^ type in iCJD with (+) and without (−) Aβ pathology. **a**-**c** age, disease duration, incubation period, and NFT pathology relative to All iCJD (**a**), GH-iCJD (**b**) and DM-iCJD (**c**); yrs.: years; mo: months. **d** and **e** ring doughnut charts visualizing genotype frequency at codon 129 (**d**) and resPrP^Sc^ type distribution (**e**) in iCJD with (iCJD-Aβ^+^) and without (iCJD-Aβ^−^) Aβ pathology; M: methionine; V: valine; T1: type 1; T2: type 2; Ti: type intermediate; Ti + 2: types intermediate +2; T1 + 2: types 1 + 2

The presence of Aβ pathology was searched for in all of the 27 iCJD and 67 sCJD cases. Cases were considered positive when at least one Aβ CP containing amyloid (Thioflavin S or Congo red positive) and/or one blood vessel harboring Aβ deposits consistent with CAA were identified in at least one histology section of a minimum of two (Table 1, Fig. 3 and Materials and Methods). Eleven of 21 iCJD cases (52%) fulfilled these selection criteria. They comprised three of the eight cases of GH-iCJD, eight of the 13 cases of DM-iCJD including the two cases of iCJDMMi (cases 9 and 21 of Table 1, Fig. 4 and Additional file 2: Table S4). Despite the relatively small size of the Aβ-positive iCJD subset, a trend could be detected when basic clinical and molecular features as well as the histopathological phenotypes were comparatively analyzed in Aβ-affected and -unaffected groups and their subsets. Subjects harboring Aβ pathology were older in each iCJD subset. Age differentials varied from ~14 to 17 years, while the incubation period was ~13 years longer in Aβ-positive GH-iCJD cases (Fig. 5). Regarding the 129 genotype, the prevalence of the MV and VV genotypes over the MM genotype observed in all iCJD cohort was slightly increased in Aβ-positive iCJD cases (57% vs. 66%) (*P* > 0.05) (Figs. 1 and 5). Furthermore, histopathological phenotypes were similarly distributed in the two iCJD groups. In Aβ-positive iCJD cases (8, 9, 10 and 27 of Table 1) with available frozen tissue the Illumina and Sanger sequencing of *APP*, *PSEN1*, and *PSEN2* genes identified no novel rare variants nor known mutations. Furthermore, *ApoE* genotyping available in Aβ-positive iCJD (8, 9, 10 and 27 of Table 1) and sCJD (65, 66 and 67 of Additional file 1: Table S2) cases underlined the lack of *ApoE-ε4/4*, and the presence of *ApoE-ε3/3* in all CJD cases with the exception of one iCJD (case 8) that had genotype *ApoE-ε2/3*.

No Aβ pathology was detected in the two GH recipients, 46 and 50 years old, free of prion disease. However, the 50-year old case may not have received the higher risk US GH produced prior 1977.

Similar Aβ pathology was also present in nine of the 67 (13%) sCJD controls (Fig. 4 and Additional file 2: Table S4). The Aβ-positive cases were on average 20 years older than the negative cases (*P* < 0.0001) and had about half the disease duration (5.5 vs. 10.4 months) (*P* = 0.029). As for 129 genotype and PrP^Sc^ type, Aβ-positive sCJD showed a higher relative representation of the MV and VV genotypes and PrP^Sc^ type 2 than the negative cases, a trend resembling that of iCJD. However, contrary to iCJD, the MV2K phenotype was not observed but the VV2 phenotype was better represented in sCJD Aβ-positive cases. Aβ pathology was also searched for in 11 non-ND cases (age 40 ± 12 years, range 25–59 years). The two oldest cases (56- and 59-year-old) were Aβ-positive harboring CP one and CAA the other (Fig. 4).

### Aβ in iCJD, sCJD and AD: Histopathological phenotype, severity and staging

All Aβ-positive iCJD cases harbored CAA. However, in about half of these subjects, CAA was accompanied by CP (CAA + CP) consistent with the presence of two phenotypes: CAA alone and CAA + CP (Additional file 2: Table S4). The mean age of the subjects harboring CAA + CP was about 15 years younger than that of the subjects with CAA alone (Tables 2 and 3). Although not statistically significant, this finding suggests that CP presence is not age-related, but more consistent with the existence of two phenotypes. In the sCJD cohort, the presence of CP alone was observed in three of the nine Aβ-positive cases along with five cases harboring CAA and one CAA + CP (Additional file 2: Table S4). Contrary to iCJD, sCJD cases harboring CP were older than those CP-free (71 vs. 64 years), possibly pointing to a relation to aging. Subpial Aβ deposition was observed in two DM- and one GH-iCJD young cases (≤ 54 years; cases 1, 8 and 16, Table 1) and in two old sCJD (> 54 years; cases 65 and 67, Additional file 1: Table S2).

As for the structural characteristics, most of the iCJD CP appeared to contain only Aβ as the only or dominant component since they reacted exclusively with Abs to Aβ; occasionally, however, they definitely also contained PrP either displaying an Aβ immunoreactive core with PrP reactive crown or the reverse or random mixtures of Aβ and PrP (Fig. 3). No significant difference was detected in the presence and brain distribution of Aβ diffuse (Thioflavin S-negative) plaques between iCJD (*N* = 4) and sCJD (*N* = 21) age-matched cases (Fig. 3 and Additional file 2: Table S5).

**Fig. 6:**
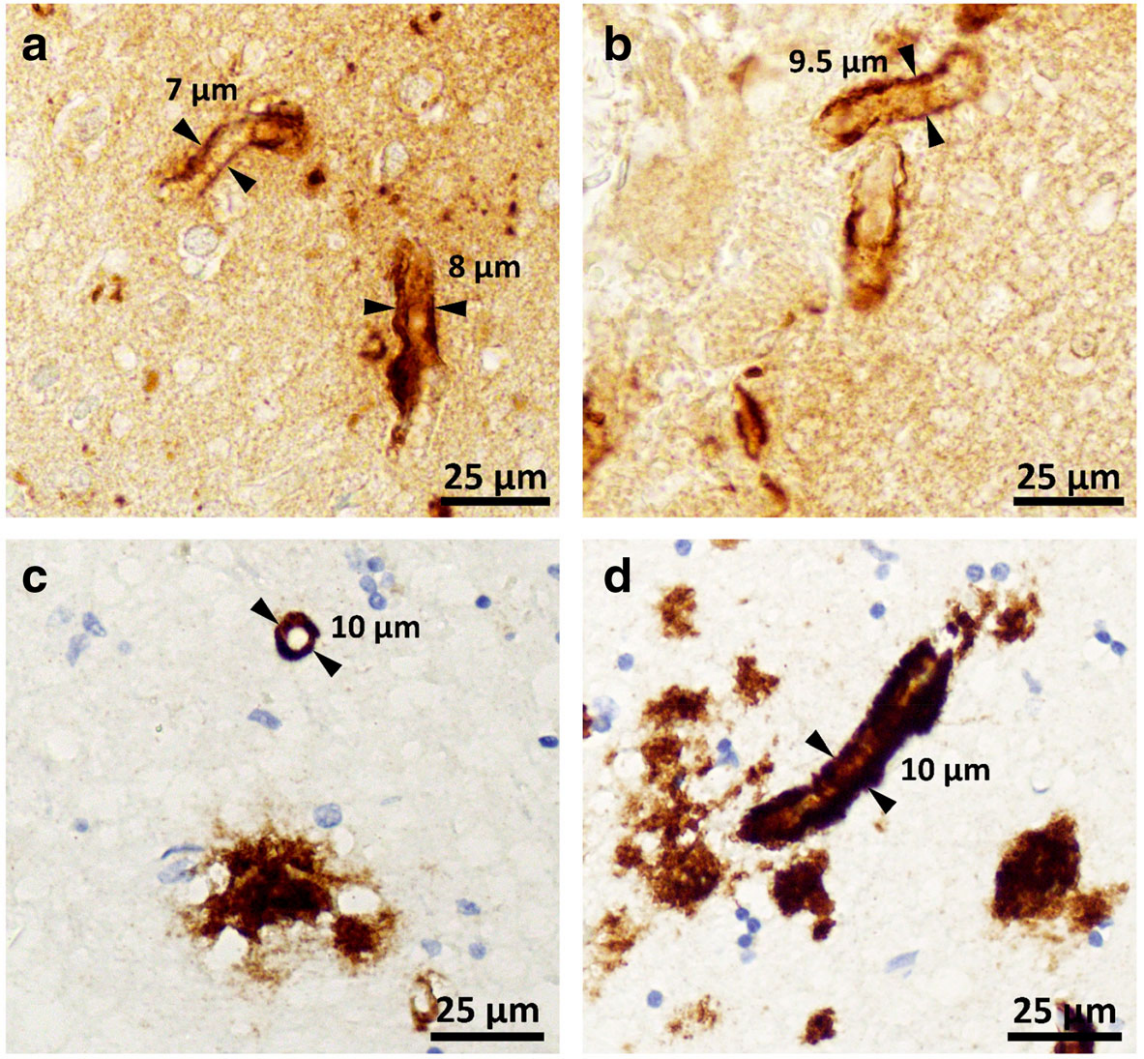
Type 1 or capillary CAA in one iCJD (**a** and **b**) and one AD (**c** and **d**). **a**-**d** Aβ deposits along the basement membrane of capillaries in the parahippocampal gyrus (**a**), molecular layer of the cerebellum (**b**) and hippocampus (**c** and **d**). The mean diameter of capillaries in longitudinal sections resulted from three (**a** and **b**) and six (**d**) measurements along the capillary whereas the diameter of the capillary in cross-section in **c** is the mean diameter calculated by the software (Image-Pro Plus)

Staging of the Aβ pathology, a measure of disease progression, was carried out in 10 Aβ-positive cases of iCJD (Tables 2 and 3). Both CAA occurring alone and CAA + CP had a widespread distribution reaching at least phase 2 in the majority of the cases (82%). The CAA severity score (0–3) ranged from 1 to 3 with a mean of 1.6, and with the exception of one case (case 1, Table 3) CAA was of type 2 (capillaries unaffected) (Table 3 and Fig. 6). As expected according to the selection criteria, amyloid was demonstrated in the CP of all five CP-harboring cases, and in the vascular Aβ deposits of seven of the 10 (70%) CAA cases examined (Tables 2 and 3, and Fig. 3). In two cases, CP were present in the cerebellum (exclusively or along with other regions) mostly populating the granule cell layer (Fig. 3). The comparative study of disease staging in iCJD with those in sCJD and AD revealed that in the sCJD cohort, only in one third of the cases Aβ pathology reached phase 2; none harbored CP in the cerebellum (Tables 2 and 3). The CAA score was 1, 62% lower than that in iCJD, but the type was exclusively 2 as in most cases of iCJD (Table 3). Amyloid was demonstrated in one of six (17%) of the CAA-affected sCJD cases. Contrary to sCJD, Aβ pathology in AD surpassed in severity the Aβ pathology observed in iCJD (Tables 2 and 3). This especially applied to CP distribution that reached phase 5 in all cases, a level of disease progression reached only by half of the cases in iCJD. Furthermore, amyloid-positive CAA was observed in all cases. As for CAA, although the difference in distribution was less striking, the mean CAA score was 2.57, about 1.6 times greater than in iCJD, but CAA was almost exclusively type 2, as in iCJD.

Morphometric characteristics of amyloid-positive CP and of blood vessels carrying Aβ deposits in CAA were compared in iCJD and AD (Additional file 6: Figure S4 and Additional file 7: Figure S5). In iCJD cerebral cortex, the CP burden was over 4 times lower (*P* < 0.004) and CP about 17% smaller than in AD (*P* < 0.0001). A small population of large CP in AD were not detected in the iCJD cohort (Additional file 6: Figure S4). In iCJD CAA-affected vessels the Aβ deposits occupied a relatively larger portion of the vessel wall than in AD (*P* < 0.0001) (Additional file 7: Figure S5).

### Tau pathology in iCJD, sCJD and AD: Lesion type, prevalence, staging and severity

NFT and DN, two prototypical lesions of tau pathology associated with typical AD, were found in 48% and 14%, respectively, of all iCJD cases (Additional file 2: Table S4). The NFT were found regardless of the presence of Aβ pathology while the DN were present in 3 cases, all harboring CP (Figs. 3 and 5). Although the NFT-positive iCJD subset was 8 years older than the NFT-free, the difference was not significant. Furthermore, the NFT prevalence in the entire iCJD cohort did not seem to change significantly as a function of age also when young (≤ 54 years) and old (> 54 years) NFT-positive iCJD subsets were assessed (47% vs. 50%). In the Aβ-positive iCJD subset, NFT and DN were found in 5/11 and 3/11 cases, respectively. The similar NFT prevalence in the Aβ-positive iCJD population when compared with that of the general iCJD population (45.5% vs. 48%) [23] suggests that the presence of Aβ pathology does not influence the prevalence of NFT.

In sCJD, prevalences of NFT and DN were 53% and 1.5% respectively, comparable to the 48% prevalence of NFT in the iCJD cohort, while the DN prevalence in sCJD compared to iCJD was much lower (Additional file 2: Table S4). The NFT prevalence appeared to be age-related given the NFT presence in 41% and 80% of the younger and older cases, respectively, and the ~ 9.5 years older age of the NFT-positive compared to the negative cases (*P* < 0.0006). Distribution and severity of NFT also were comparable to those of the corresponding iCJD cohort and so was the age relation (Additional file 2: Table S6). Much higher burdens of both NFT and DN with no evidence of age effect were observed in all AD cases. However, brain distribution was comparable to that of iCJD.

## Discussion

We report on 21 cases of iCJD including GH-iCJD and DM-iCJD subsets collected in Australia, France, Italy and the US, 11 (52%) of which harbored significant Aβ pathology. Despite the diversity of their geographic origin, our iCJD cases were remarkably similar concerning the 129 genotype and CJD histopathological phenotype distributions, and the features of the associated Aβ pathology.

For DM-iCJD, the similarities may reflect the use of the same Lyodura product in all these countries. In contrast, the cases of GH-iCJD were all from the US and can only be compared with the similar cohorts studied in the UK and France, where methods of GH production differed from those in the US (SH and JPB, personal communication).

Genotype and phenotype distribution in our iCJD cohort showed a relatively high prevalence of MV and VV over MM codon 129 genotypes when compared to sCJD as also observed in previous studies [54, 55]. Furthermore, we observed two iCJD with the MMi phenotype that has been shown to originate from adaptation of sCJDVV2 or sCJDMV2K to the 129MM background of the recipient [39, 42, 54, 59]. The finding that this event, initially described in Japanese DM-iCJD, is also observed in our international iCJD cohort as well as the UK cohort, and thus is widespread, suggests that 129VV and 129MV individuals may have been preferentially selected as donors in several countries. Alternatively, subjects with 129MV and 129VV genotypes might be more susceptible to acquire CJD than the carriers of the 129MM genotype, in whom the sCJDMM1 phenotype might be difficult to reproduce requiring strain adaptation [13, 46, 69]. The ~2 times longer incubation period in GH-iCJD than DM-iCJD cases resembles the longer incubation period associated with the peripheral (e.g., in the peritoneal cavity) vs. central (e.g., intracerebral) inoculations of prions or Aβ seeds in Tg mouse models for these proteinopathies [20, 36]. Concerning the Aβ pathology, iCJD-affected subjects had CAA accompanied by parenchymal Aβ CP in five cases and subpial deposits in three. The Aβ-positive iCJD subset also had a relatively young age (mean 50.5 years, range 26–75 years) along with the relative severity and widespread distribution of CAA [66]. Furthermore, with only one exception, CAA was type 2, i.e. did not affect cerebral capillaries unlike type 1 [66]. Since CAA type 1 has been directly associated with AD severity, including tau pathology, age, and with *ApoE-ε4* allele frequency, the predominance of type 2 CAA in iCJD is not surprising given the relatively younger age and short duration of this condition [52, 62].

Aβ pathology was examined in age-group matched cohorts of sCJD and of non-ND used as controls to rule out the possibility that Aβ deposition in iCJD was either related to aging or resulted from the concomitant aggregation of prion or prion-like proteins by a cross-seeding mechanism. Furthermore, type and severity of the iCJD Aβ pathology was compared with that of AD. The 13% prevalence of Aβ pathology in the sCJD cohort (mean age 50 years, range 24–79 years) was 4 times lower than that in iCJD (52%) even though the Aβ-positive sCJD subset was 17 years older. In the non-ND cohort (age 40 ± 12 years, range 25–59 years), the 2 (18%) Aβ-positive cases were 56 and 59 years of age and the oldest of this cohort. When all Aβ-positive subsets were considered in the 20–54 years range, the prevalence of Aβ pathology in iCJD, sCJD and non-ND populations was 41%, 2% and 0%, respectively. Furthermore, the Aβ pathology in iCJD cases was more severe than sCJD, and showed a significantly more widespread CP and CAA distribution and a trend toward a significantly higher CAA severity score. On the contrary, Aβ pathology in AD was dramatically more severe than in Aβ-positive iCJD with regard to number, size and distribution of CP as well as of CAA in neocortical regions, even though the AD cohort used as control had a relatively young age (64 ± 8 years). These features clearly distinguish the Aβ pathology of iCJD from typical AD. Although we cannot rule out that the Aβ pathology might have evolved toward an AD phenotype had the Aβ positive iCJD patients lived longer and not died of CJD this possibility is unlikely since no significant differences were found between the UK GH-iCJD and hGH recipients free of CJD. Even though we used a different criterion for Aβ plaque selection, our findings on Aβ pathology do not significantly differ from those reported in five previous studies [19, 23, 31, 37, 53], which collectively examined 95 cases of iCJD, including 67 GH-iCJD and 28 DM-iCJD. However, the 54% prevalence of the Aβ pathology in UK GH-iCJD is higher than the 37.5% we observed. Similarly, the 18 year mean incubation period of UK GH-iCJD is significantly shorter than that of US cases (28 years) (*P* < 0.004). These differences may relate to the relatively high prevalence of UK GH-iCJD cases (~4.2%) compared to the 1.2% prevalence among US recipients of pre-1977 produced human GH (hGH) as well as to variations in selecting pituitary donors and in protocols of GH purification [2, 4, 7, 54]. Regardless of the causes, the higher prevalence of the Aβ pathology, the significantly lower mean incubation period and the more than a decade younger age differential of UK Aβ-positive GH-iCJD cases, suggest that GH used in UK had a higher infectious dose not only of PrP^Sc^ but also of Aβ seeds. Remarkably, despite these differences, the Aβ phenotype in US and UK was similarly characterized by CAA alone or co-existing with CP. The small subset of Aβ-positive GH-iCJD cases harboring parenchymal Aβ deposits reported by Ritchie and co-workers (2017a) presumably included diffuse plaques, which may also account for the higher prevalence of positive Aβ pathology in UK GH-iCJD. Furthermore, CAA reached similar severity scores in US (1.6) and UK (2) and type 2 CAA markedly predominated in both countries [37, 53]. The very low prevalence of the Aβ pathology (4%) in French GH-iCJD cases compared to the US and UK GH-iCJD cases has been explained possibly by the few years shorter incubation period in French GH-iCJD and to differences in GH preparations [19]. The prevalence of the Aβ pathology in DM-iCJD reported here (61.5%) is similar to that of the previous studies combined (69%) but differs from the 81% prevalence reported by Hamaguchi et al. (2016). This difference may depend on the older age of the Japanese cohort compared to those examined by us and Frontzek et al. (2016) (10 and 16 years, respectively). The co-occurrence of CAA and Aβ parenchymal deposits was observed in all the cases of Frontzek and co-workers (2016) but in only half of ours (CAA occurred alone in the others). This discrepancy along with the aforementioned discrepancy of UK GH-iCJD cases are likely due to our different criteria of Aβ plaque validation. Unlike the five previous studies that also accepted diffuse plaques, we validated only CP (i.e. Aβ plaques that contained amyloid) to better distinguish Aβ pathology associated with iCJD from that related to aging [19, 23, 31, 37, 53]. The exclusion of the plaque amyloid requirement would have increased the the Aβ-positive iCJD cases from 11 to 13 and increased by 9 the number of Aβ-positive sCJD cases (Additional file 2: Table S5 and data not shown). The CP requirement in the Aβ pathology of iCJD might establish a qualitative difference in the Aβ phenotype between iCJD and sCJD in younger populations. Nonetheless, this and previous studies show that CAA, with or without CP, is the distinctive histopathological phenotype of Aβ deposition in iCJD [19, 23, 31, 37, 53]. Our study taking advantage of the direct comparison, also shows that the Aβ phenotype is similar in GH- and DM-iCJD.

Because tau pathology is considered a consistent but secondary feature of AD, we also searched for the presence of the two most common tau related lesions, NFT and DN [33, 34, 57]. NFT were present in Aβ-negative and Aβ-positive iCJD, as well as in sCJD controls with similar prevalences (45–53%), and they were age-related. Occurrence of NFT in absence of Aβ plaques was previously shown in sCJD and was considered as “primary age-related tauopathy” in the elderly [17, 44, 51]. Overall, the NFT prevalence in cases with less than 52 years were similar in iCJD (44%) and sCJD (40%) cases, and did not differ from that reported in a large population of unselected individuals of similar age (41%) [6]. Neocortical DN were observed in only three iCJD cases and one case of sCJD where they were occasionally associated with CP, as previously reported [53]. In contrast, NFT and DN were consistently present in the AD cohort. These findings indicate that tau pathology is (i) a non-obligatory component of iCJD Aβ phenotype, (ii) likely develops independently from the Aβ pathology in iCJD, and (iii) further distinguishes iCJD Aβ pathology from AD.

Further important questions raised by our and previous studies are the origin of Aβ seeding, how Aβ seed reaches the brain, and whether Aβ-seeded diseases are contagious.

In hGH recipients, Ritchie and colleagues [53] convincingly showed that Aβ deposition occurs in the absence of prion pathology and the phenotype associated with the Aβ deposition remains similar to that of Aβ-positive iCJD cases, suggesting that Aβ deposition is a primary co-pathology in GH-iCJD and that Aβ and PrP^Sc^ seeding processes occur independently.

Although we occasionally have observed Aβ-PrP mixed plaques supporting the possibility of co-seeding, the brunt of the two pathologies were anatomically segregated: Aβ deposition affected mostly vessel walls while PrP^Sc^ affected exclusively the brain parenchyma. Moreover, the fact that Aβ-positive iCJD is associated with different CJD subtypes argues against cross-seeding of Aβ by a specific prion strain. Additional support to the independent seeding of Aβ in DM-iCJD comes from two other observations. First, Aβ deposits occurred in the dura graft but not in the patient’s original dura [43], secondly, the distribution of Aβ deposits is consistent with the propagation through the brain of Aβ pathology originating from the dura graft while the distribution of PrP^Sc^ pathology is uniform [31, 43]. These findings point to the dura graft as the source of the Aβ seed and to a different tempo of Aβ and PrP^Sc^ propagation further strengthening the notion that in iCJD PrP^Sc^ and Aβ are independent pathologies. In GH-iCJD, the Aβ seed is likely to propagate from the site of cutaneous injection to the brain. Experimental data have definitely provided the proof of principle that human Aβ seed may reach the brain causing Aβ amyloidosis following systemic inoculation [20]. Remarkably, a predominantly vascular distribution of the Aβ deposits consistent with Aβ-CAA was noted in these experiments [20].

## Conclusions

Although our and previous studies do not rule out important roles for other factors contributing to Aβ pathology in iCJD such as hGH deficiency or the DM-associated neurosurgical procedures, they point to human-to-human transmission of Aβ with mechanisms similar to those of PrP^Sc^ [19, 23, 31, 37, 53].

## Additional files

Additional file 1: Table S1. Age, disease duration and incubation period in iCJD and sCJD divided into three groups according to age at death. **Table S2.** Demographic data and molecular subtypes of 67 sCJD cases from the countries providing iCJD cases used as age-group matched controls in assessing prevalence of Aβ and tau pathologies. **Table S3.** Demographic and medical data on iCJD and sCJD used as controls. (DOCX 37 kb)

Additional file 2: Table S4. Prevalence of Aβ and tau pathologies in iCJD and sCJD used as controls. **Table S5.** Aβ diffuse plaque staging in cases of iCJD and sCJD not associated with Aβ CP pathology. **Table S6.** Brain distribution and severity of NFT and DN in iCJD and control cases of sCJD and AD. (DOCX 49 kb)

Additional file 3: Figure S1. Kaplan–Meier estimates of iCJD and sCJD controls. **A:** survival graphs representing whole iCJD cohort and its GH- and DM-iCJD subsets as well as sCJD cases. **B**: survival graphs of 21 iCJD and all sCJD divided into two groups according to age: 54 years or younger (≤54y), and older than 54 years (> 54y). Median survivals are indicated on the X-axis in **A** and **B** (arrowheads). Significant differences in median survivals, determined by the log rank (Mantel-Cox), were found between GH-iCJD and sCJD (P=0.0017) (**A**) or DM-iCJD ≤ 54y and sCJD ≤ 54y (P=0.0027) (**B**). (PDF 2348 kb)

Additional file 4: Figure S2. Histopathology of an atypical US iCJD (case 5, Table 1). **A**-**C**: HE staining. **D** and **E**: PrP immunohistochemistry. **A**: spongiform degeneration (SD) with large and confluent vacuoles typical of the sCJDMM2 subtype, but limited to layer II of the frontal cortex, and severe neuronal loss and gliosis affecting all layers. **B**: high magnification of the area highlighted in **A** showing SD with large vacuoles. **C**: severe loss of granule cells and gliosis of the cerebellum; Grl. L: granular layer; Mol. L: molecular layer. **D** and **E**: diffuse or “synaptic” PrP immunostaining in the frontal cortex (**D**), and molecular and granular layers of the cerebellum (**E**); **A**-**C**: HE staining. **D** and **E**: PrP immunohistochemistry with Ab 3F4. (PDF 30981 kb)

Additional file 5: Figure S3. WB profile of resPrP^Sc^ from iCJD and sCJD controls using different WB systems for PrP detection. BHs from the frontal cortex (lanes 8-10) and cerebellum (lanes 1-7) treated with 100 U/ml PK (~2000 μg/ml) were probed with indicated Abs. PrP bands were resolved in 15% Tris-HCl, 8.7 cm-long (**A**-**C** and **E**) and 20 cm-long (**D**) gels, and visualized with near-infrared LI-COR system (**A**-**D** and **E**(**i**)) or chemiluminescence (**E**(**ii**) and **E**(**iii**)). **A**: profiles of resPrP^Sc^ from CJD obtained with an 8.7 cm-long gel are similar to those obtained with the 20 cm-long gels in Fig. 2. The unglycosylated resPrP^Sc^ ~21 kDa of type 1 (21), ~20 kDa of type i (i), ~19 kDa of type 2 (2) and ~18 kDa (18) are indicated. **B**: the ~20 kDa of type i (i) and ~21 kDa of type 1 (21) (lanes 3-8), but not the ~19 kDa band of type 2, immunoreact with Ab 12B2. Profile of resPrP^Sc^ from iCJDMMi (lane 5) is indistinguishable from that of CJDMVi+2 (lanes 3 and 4), but differs from those of iCJDMM1 and sCJDMM1 (lanes 6-8), as the ~21 kDa band predominates in these three conditions. **C**: only ~19 kDa (type 2), but not ~21 kDa (type 1) and ~20 kDa (type i) immunoreacted with Tohoku-2 (To-2). An ~18 kDa (18) band in CJDVV2 and CJDMVi+2, and an unidentified lower size fragment (large arrow) in all cases harboring type 2 were also detected. A non-specific band (indicated by three asterisks) was present in all tested samples. **D**: high resolution gel electrophoresis in 20-cm long gel revealing ~21 kDa (21) and the ~20 kDa band (i and arrowhead). **E**(**i**): enhanced image of dashed area in **A** showing the bands ~21 kDa (21), ~20 kDa (i and arrowhead), and ~19 kDa (19; lane 4). The detection of the resPrP^Sc^ ~20 kDa band in addition to the prominent ~21 kDa fragment in CJDMM1 is most likely due to our use of a high resolution electrophoretic systems. **E**(**ii**): profiles of unglycosylated resPrP^Sc^ from the same cases in **E**(**i**) visualized on film by chemiluminescence**.** Note that the three CJDMM1 (lanes 6-8) show only ~21 kDa bands. **E**(**iii**): the ~20 kDa and ~21 kDa bands visible in **B** (lanes 3-5) resolve as a single band of ~21 kDa (lanes 4 and 5) when visualized on film by chemiluminescence. (PDF 6122 kb)

Additional file 6: Figure S4. Quantitative estimates of Thioflavin S-positive Aβ CP affecting the cerebral cortex in subjects with iCJD and sporadic AD (sAD). **A**: the density of Aβ CP, expressed as the percentage of cerebral cortex area occupied, was 5 times greater in subjects with sAD than in iCJD. **B**: size of Aβ CP (N=500), expressed as diameter, was greater in patients with sAD than in those with iCJD. **C** and **D**: representative microscopic fields showing fewer and smaller CP (arrows) in iCJD (**C**) than sAD (**D**) where cluster of very large CP (dashed square) could be detected. Bar graphs are expressed as mean ± standard error of the mean (SEM) in **A** or as mean ± standard deviation in **B**. Student's t-test (two-tailed). (PDF 3338 kb)

Additional file 7: Figure S5. Size of blood vessels examined and semiquantitative evaluation of the percentage of the vessel wall perimeters occupied by Aβ deposits in subjects with iCJD and sporadic AD (sAD). **A**: scatter plot showing the similar sizes of the blood vessels from the subarachnoid spaces of frontal, occipital and cerebellar regions examined for the semiquantitative determinations made in **B**; size of individual vessels is measured as perimeter. **B**: the percentage of the blood vessel wall occupied by Aβ was significantly greater in iCJD than sAD. Bar graphs are expressed as mean±SEM. Student's t-test (two-tailed). (PDF 228 kb)

## Abbreviations

Aβ: amyloid β
AD: Alzheimer’s disease
*APP*: amyloid precursor protein
*PSEN1*: presenilin 1
*PSEN2*: presenilin 2
*ApoE*: Apolipoprotein E
Ab: antibody
GH: growth hormone
hGH: human GH
DM: dura mater
DM_L_: Lyodura® brand DM
DM_T_: Tutoplast® brand DM
CJD: Creutzfeldt-Jakob disease
sCJD: sporadic CJD
iCJD: iatrogenic CJD
GH-iCJD: GH-associated iCJD
DM-iCJD: DM-associated iCJD
p-tau: highly phosphorylated tau
PrP^C^: normal or cellular prion protein (PrP)
PrP^Sc^: scrapie PrP
resPrP^Sc^: proteinase K-resistant PrP^Sc^
PK: proteinase K
BH: brain homogenate
WB: Western blot
M: methionine
V: valine
T1: type 1
T2: type 2
T1+2: type 1+2
Ti: type intermediate
Ti+2: type intermediate+2
CAA: cerebral amyloid angiopathy
CP: core plaques
NFT: neurofibrillary tangles
DN: dystrophic neurites
HE: hematoxylin-eosin
Non-ND: non-neurodegenerative disorders
NPDPSC: National Prion Disease Pathology Surveillance Center
