# Supplementary material for: Iatrogenic Creutzfeldt-Jakob disease with Amyloid-β pathology: an international study
Source: Acta Neuropathol Commun. 2018 Jan 8;6:5. doi: 10.1186/s40478-017-0503-z (PMC5759292; doi:10.1186/s40478-017-0503-z)
Supplement: Supplementary file 1 — Age, disease duration and incubation period in iCJD and sCJD divided into three groups according to age at death. Table S2. Demographic data and molecular subtypes of 67 sCJD cases from the countries providing iCJD cases used as age-group matched controls in assessing prevalence of Aβ and tau pathologies. Table S3. Demographic and medical data on iCJD and sCJD used as controls. (DOCX 37 kb) [file 40478_2017_503_MOESM1_ESM.docx]

| Age group | ≤ 54y and > 54y | | | | ≤54y | | | >54y | |
| --- | --- | --- | --- | --- | --- | --- | --- | --- | --- |
| **Diagnosis**  N cases | **All-iCJD**  (27) | **GH-iCJD**  (13) | **DM-iCJD**  (14) | **sCJD**  (67) | **GH-iCJD**  (13) | **DM-iCJD**  (10) | **sCJD**  (47) | **DM-iCJD**  (4) | **sCJD**  (20) |
| **Age at death** (y)  Range (y) | 41.9±14.2^a^  (21-75) | 40.9±8.5  (23-54) | 43.1±18  (23-75) | 49.9±11.4  (24-79) | 40.9±8.5  (23-54) | 33.4±9.2  (23-50) | 43.8±6.4  (24-52) | 67.5±6.6  (62-75) | 64.2±6.4  (55-79) |
| **Disease duration** (mo)  Range (mo) | 8.5±7  (2-26) | 10.8±8  (2-26) | 6.8±6.5  (2-27) | 9.8±14.1  (1-75) | 10.8±8.1  (2-26) | 8.2±7.3  (3.5-27) | 11.8±16  (1-75) | 3.2±1  (2-4) | 5.1±6  (1-28) |
| **Incubation period** (y)  Range (y) | 19.4±11.3  (4-43) | 27.7±9.5  (10-43) | 12.4±7.1  (4-25) | na | 27.7±9.5  (10-43) | 14.2±6.6  (6-25) | na | 7.8±6.8  (4-18) | na |

**Table S1** Age, disease duration and incubation period in iCJD and sCJD divided into three groups according to age at death

^a^Mean±SD; y: years; mo: months na: not available.

**Table S2** Demographic data and molecular subtypes of 67 sCJD cases from the countries providing iCJD cases used as age-group matched controls in assessing prevalence of Aβ and tau pathologies

Case Country Age Disease Codon 129 PrP^Sc^ Case Country Age Disease Codon 129 PrP^Sc^

number (years) durat. (mo) genotype type number (years) durat. (mo) genotype type

1 United States 30 3 MM 1 35 United States 50 1 MM 1

2 30 75 VV 1 36 50 2 MM 1

3 33 3 MM 1 37 51 11 MM 1+2

4 35 13 VV 1 38 52 39 MV 2

5 37 3 MV 1 39 56 28 MV 1+2

6 38 4 MM 1 40 56 1 MM 1

7 38 12 VV 1 41 60 3 MM 1

8 39 7 MM 1 42 61 2 MM 1

9 43 3 MM 1 43 62 2 MM 1

10 43 2 MM 1 **44**^a^ 64 1 MM 1

11 43 6 MM 1 45 Australia 55 3.5 MM 1

12 44 53 MM 1 46 57 5 na 2

13 44 5 MM 1 47 63 2 MV 1

14 44 3 MM 1 48 63 2 MM 1

15 44 7 VV 1+2 49 France 24 62 VV 1+2

16 45 5 VV 1+2 50 34 19 VV 1

17 45 24 MV i+2 51 34 27 MM 2

18 45 16 MM 1 52 43 17 MM 1+2

19 45 3 MM 1 53 43 6 MM 1

20 46 2 MM 1 54 49 7 MM 1

21 47 3 MV 1 **55** 49 4 MM 1

22 47 6 MV i+2 **56** 63 4 MM 1

23 47 1 MM 1 **57** 63 3 MV 1

24 47 23 MM 1 **58** 71 8 VV 2

25 47 4 MM 1 **59** 71 9 MV 2

26 48 2 MM 1 60 Italy 43 3.5 MM 1

27 48 1 MM 1 61 48 17 MM 1

28 48 3 MV 1 62 65 1.5 MM 1

29 49 1 MM 1 63 65 2.5 MM 1

30 50 3 MM 1 64 66 3 MM 1

31 50 2 MM 1 **65** 71 10 VV 2

32 50 9 MM 1 **66** 74 4.5 VV 2

33 50 21 MV i+2 **67** 79 6 VV 2

34 50 10 MV i+2

^a^Numbers in bold indicate sCJD cases with Aβ-positive pathology; durat.: duration; mo: months; na: not available.

**Table S3** Demographic and medical data on iCJD and sCJD used as controls

**Variable iCJD (%)**^a^ **sCJD (%)**^a^ **P value**

Cases (all male) 20 (71) 40 (60) NS*

Age (years) 41.4±14.2^b^ 49.9±11.4^b^ 0.002**

Duration (months) 9.4±8.7^b^ 9.8±14.1^b^ NS**

Intracranial tumor 8 (33) 2 (3) 0.004

Head trauma 8 (36) 2 (3) <0.001

Other Neuro/Psychiatric 11 (46) 3 (5) <0.001

conditions (including HIV)

Brain surgery 13 (59) 1 (2) <0.001

Family history of dementia 3 (15) 9 (14) NS

^a^% of all cases with available data; ^b^mean ± standard deviation; *Chi-square, 2-sided analyses; **Student's t-test, 2-tailed; other analyses were determined by Fisher’s exact test; NS: not significant.
