# Supplementary material for: Iatrogenic Creutzfeldt-Jakob disease with Amyloid-β pathology: an international study
Source: Acta Neuropathol Commun. 2018 Jan 8;6:5. doi: 10.1186/s40478-017-0503-z (PMC5759292; doi:10.1186/s40478-017-0503-z)
Supplement: Supplementary file 2 — Prevalence of Aβ and tau pathologies in iCJD and sCJD used as controls. Table S5. Aβ diffuse plaque staging in cases of iCJD and sCJD not associated with Aβ CP pathology. Table S6. Brain distribution and severity of NFT and DN in iCJD and control cases of sCJD and AD. (DOCX 49 kb) [file 40478_2017_503_MOESM2_ESM.docx]

**Table S4** Prevalence of Aβ and tau pathologies in iCJD and sCJD used as controls

| **Variable** | iCJD  (N=21) | sCJD  (N=67) | GH-  iCJD≤54y  (N=8) | sCJD  ≤54y  (N=47) | DM-iCJD≤54y  (N=9) | sCJD  ≤54y  (N=47) | DM-  iCJD>54y  (N=4) | sCJD  >54y  (N=20) |
| --- | --- | --- | --- | --- | --- | --- | --- | --- |
| **Aβ prevalence** |  |  |  |  |  |  |  |  |
| Based on (A)-(C) | 52^a^  (11/21^b^) | 13  (9/67) | 37.5  (3/8) | 2  (1/47) | 44  (4/9) | 2  (1/47) | 100  (4/4) | 40  (8/20) |
| **Aβ phenotypes** |  |  |  |  |  |  |  |  |
| (A) CP | 0  (0/11^d^) | 33  (3/9) | 0  (0/3) | 0  (0/1) | 0  (0/4) | 0  (0/1) | 0  (0/4) | 37.5  (3/8) |
| (B) CAA | 54.5  (6/11) | 55.5  (5/9) | 33  (1/3) | 100  (1/1) | 50  (2/4) | 100  (1/1) | 75  (3/4) | 50  (4/8) |
| (C) CAA+CP^e^ | 45.5  (5/11) | 11  (1/9) | 66  (2/3) | 0  (0/1) | 50  (2/4) | 0  (0/1) | 25  (1/4) | 12.5  (1/8) |
| (D) Subpial | 30  (3/10^g^) | 22  (2/9) | 33  (1/3) | 0  (0/1) | 50  (2/4) | 0  (0/1) | 0  (0/3^g^) | 25  (2/8) |
| **Tau prevalence** |  |  |  |  |  |  |  |  |
| NFT | 48  (10/21^c^) | 53  (35/66^h^) | 88  (7/8) | 41  (19/46^h^) | 11  (1/9) | 41  (19/46^h^) | 50  (2/4) | 80  (16/20) |
| DN | 14  (3/21) | 1.5  (1/66^h^) | 12.5  (1/8) | 0  (0/46^h^) | 11  (1/9) | 0  (0/46^h^) | 25  (1/4) | 5  (1/20) |

^a^Percentage of cases with Aβ or tau pathology; ^b,c^Cases with positive ^b^Aβ or ^c^tau pathology/total cases examined; ^d^Cases with the Aβ phenotype/total Aβ-positive CJD cases; ^e^Aβ phenotype with CAA and CP; ^g^Data on subpial Aβ not available in one iCJD case; ^h^Data on NFT and DN not available in one sCJD; Aβ prevalence: (i) iCJD vs. sCJD, P<0.0003*; (ii) GH-iCJD≤54y vs. sCJD≤54y, P<0.009; (iii) DM-iCJD≤54y vs. sCJD≤54y, P<0.002; Tau prevalence: (iv) NFT, GH-iCJD≤54y vs. sCJD≤54y, P<0.03; (v) DN, iCJD vs. sCJD, P<0.05; *Chi-square test; other analyses were determined by Fisher’s exact test.

**Table S5** Aβ diffuse plaque staging in cases of iCJD and sCJD not associated with Aβ CP

pathology

Case Age Disease Cod. 129 PrP^Sc^ **Phase 1** **Phase 2** **Phase 3-4** **Phase 5** Thiofl^a^

number (y) durat. (mo) genotype type Neocortex Hippoc. Subcortical Cerebellum

**iCJD**

1 (3)^b^ 33 6 VV na ++^c^ ++ - - -

2 (10) 54 2 MM 1 + - - - -

3 (22) 62 4 VV na +^d^ - - - -

4 (23) 71 4 MV na +++^d^  ++ -^e^ - -

mean±SD 55±16 4±2 50%^f^ 50% 0% 0% 0%

**sCJD**

1 (51)^g^ 34 27 MM 2 -^d^ + - - nt

2 (4) 35 13 VV 1 + - - - -

3 (14) 44 3 MM 1 +++ + - - -

4 (16) 45 5 VV 1+2 + - - - -

5 (21) 47 3 MV 1 + - na - na

6 (25) 47 4 MM 1 + - - - -

7 (27) 48 1 MM 1 ++++ - - - -

8 (61) 48 17 MM 1 + - - - nt

9 (29) 49 1 MM 1 ++++ - - na -

10 (32) 50 9 MM 1 +++ - - - -

11 (34) 50 10 MV i+2 ++++ - - - -

12 (40) 56 1 MM 1 +++ + - - -

13 (39) 56 28 MV 1+2 +++ - + - -

14 (41) 60 3 MM 1 + - - - -

15 (43) 62 2 MM 1 ++ - - - -

16 (56) 63 4 MM 1 +++^d^ + - - -

17 (47) 63 2 MV 1 ++++ ++ - - nt

18 (44) 64 1 MM 1 + - - - -

19 (64) 66 3 MM 1 ++++ ++ - - nt

20 (58) 71 8 VV 2 +++^d^  - - - -

21 (66) 74 4.5 VV 2 ++++ + - - -

mean±SD 53±11 7±8 63% 21% 2% 0% 0%

P value NS^h^ NS^i^ NS^i^ NS^i^ NS^i^

Phases 1 to 5 refer to Aβ plaques deposition in various brain regions reflecting disease progression according to Thal et al [63]; ^a^Thioflavin S (Thiofl) staining performed in brain regions with Aβ plaque pathology; ^b,g^Numbers in parenthesis in the first column refer to numerals used in ^b^Table 1 and ^g^Supplementary Table 2; ^c^Minus and plus signs indicate absence (-) or presence (+) of Aβ pathology affecting one (+), two (++), three (+++) or four (++++) of the brain regions that characterize each phase; Phase 1: frontal, temporal, parietal and occipital cortical regions; Phase 2: hippocampus and entorhinal cortex; Phase 3-4: striatum, thalamus and midbrain; Phase 5: cerebellum; ^d^Refers to two iCJD and three sCJD cases with missing parietal cortex; ^e^Refers to one iCJD with missing thalamus; ^f^Percentage of brain regions with Aβ diffuse plaques; ^h^Chi-square, 2-sided analyses; ^i^Fisher’s exact test; Hippoc.: hippocampal formation (hippocampus and entorhinal cortex); y: years; durat.: duration; Cod.: codon; na: not available; nt: not tested; NS: not significant; SD: standard deviation.

**Table S6** Brain distribution and severity of NFT and DN in iCJD and control cases of sCJD and AD

NFT DN

Case Age Disease Codon 129 PrP^Sc^ Hippoc. Neocortex Subcort. *Severity*^a^ Neocortex *Severity*^a^

number (y) durat. (mo) genotype type

**iCJD**

1 (1)^b^ 26 5 na na -^c^ -^d^ -^e^ na ++^d^ mild

2 (3) 33 6 VV na - ++ - mild - -

3 (4) 39 17 MV na - + +++ mild - -

4 (2) 39 4 MM 1 - + - mild - -

5 (6) 41 2 MM 1 ++ + +^d^ mild - -

6 (7) 43 26 MV i+2 ++ +^e^ -^d^ mild -^e^ -

7 (8) 44 18 MV i+2 - + + mild ++++ mild

8 (9) 51 14 MM i - - ++ mild - -

9 (10) 54 2 MM 1 + + - mild - -

10 (22) 62 4 VV na + -^f^ - mild -^f^ -

11 (17) 62 2 na na - - - na ++++ na

12 (23) 71 4 MV na + -^f^ - mild -^f^ -

mean±SD 47±13 9±8 29% 18% 22% 23%

**sCJD**

1 (1)^g^ 30 3 MM 1 + - - mild - -

2 (51) 34 27 MM 2 + -^f^ - mild -^f^ -

3 (4) 35 13 VV 1 - + - mild - -

4 (11) 43 6 MM 1 - - + mild - -

5 (53) 43 6 MM 1 + -^f^ - mild -^f^ -

6 (12) 44 53 MM 1 - + - mild - -

7 (14) 44 3 MM 1 - + -^d^  mild - -

8 (19) 45 3 MM 1 - - + mild - -

9 (16) 45 5 VV 1+2 + + - mild - -

10 (26) 48 2 MM 1 - ++ + mild - -

11 (27) 48 1 MM 1 ++ +++ +++ moderate - -

12 (29) 49 1 MM 1 - + - mild - -

13 (55) 49 4 MM 1 + -^f^ - mild -^f^ -

14 (30) 50 3 MM 1 - ++ - mild - -

15 (33) 50 21 MV i+2 + - -^d^ mild - -

16 (34) 50 10 MV i+2 - + +^d^ mild - -

17 (36) 50 2 MM 1 - + - mild - -

18 (37) 51 11 MM 1+2 ++ + + mild - -

19 (38) 52 39 MV 2 ++ - - mild - -

20 (39) 56 28 MV 1+2 + + - moderate - -

21 (40) 56 1 MM 1 + + - mild - -

22 (41) 60 3 MM 1 + +++ +++ moderate - -

23 (42) 61 2 MM 1 + - - mild - -

24 (43) 62 2 MM 1 ++ + - mild - -

25 (56) 63 4 MM 1 + +^f^ + mild -^f^ -

26 (57) 63 3 MV 1 ++ +^f^ - mild -^f^ -

27 (47) 63 2 MV 1 + - - mild - -

28 (44) 64 1 MM 1 + + - mild - -

29 (62) 65 1.5 MM 1 + - - mild - -

30 (64) 66 3 MM 1 + - - mild - -

31 (58) 71 8 VV 2 + -^f^ - mild -^f^ -

32 (59) 71 9 MV 2 ++ ++^f^ - mild -^f^ -

33 (65) 71 10 VV 2 ++ - - mild - -

34 (66) 74 4.5 VV 2 + - - mild - -

35 (67) 79 6 VV 2 + + - mild + mild

mean±SD 54±12 9±12 46% 20% 12% 1%

P value^h^ NS NS NS <0.0001

**AD**

1 56 60 na na ++ ++++ +++ severe ++++ severe

2 56 60 na na ++ ++++ +++ severe ++++ severe

3 59 na na na ++ ++++ +++ severe ++++ severe

4 60 130 na na +^d^ ++++ +++ severe ++++ severe

5 70 66 na na ++ ++++ +++ severe ++++ severe

6 72 111 na na ++ ++++ +++ severe ++++ severe

7 74 22 na na ++ ++++ +++ severe ++++ severe

mean±SD 64±8 75±39 100% 100% 100% 100%

P value^i^ <0.0001 <0.0001 <0.0001 <0.0001

^a^Severity of NFT and DN refers to the density of NFT or DN in the microscopic field (area: 1.3 x 1.0 mm^2^, using a 10X objective) harboring the highest density of NFT or DN; degrees of severity are defined as: mild (≤ 10 NFT or DN), moderate (>10 to <30 NFT or DN), and severe (≥30 NFT or DN); ^b,g^Numbers in parenthesis in the first column refer to numerals used in ^b^Table 1 and ^g^Supplementary Table 2; ^c^Minus and plus signs indicate absence (-) or presence (+) of NFT or DN pathology affecting one (+), two (++), three (+++) or four (++++) brain regions of hippocampal formation, neocortex (frontal, temporal, parietal and occipital cortices) and subcortical compartment (striatum, thalamus and midbrain); ^d^Refers to three iCJD, three sCJD and one AD with one missing brain region; ^e^Refers to two iCJD patients with two missing brain regions; ^f^Refers to two iCJD and seven sCJD cases with missing parietal cortex; ^h,i^Fisher’s exact test comparing iCJD to ^h^sCJD or ^i^AD; Hippoc.: hippocampal formation (hippocampus and entorhinal cortex); Subcort.: subcortical regions (striatum, thalamus and midbrain); y: years; mo; months; durat.: duration; na: not available; NS: not significant; SD: standard deviation.
