# Supplementary figures and images for: Iatrogenic Creutzfeldt-Jakob disease with Amyloid-β pathology: an international study
Source: Acta Neuropathol Commun. 2018 Jan 8;6:5. doi: 10.1186/s40478-017-0503-z (PMC5759292; doi:10.1186/s40478-017-0503-z)

# Supplementary Figure 1

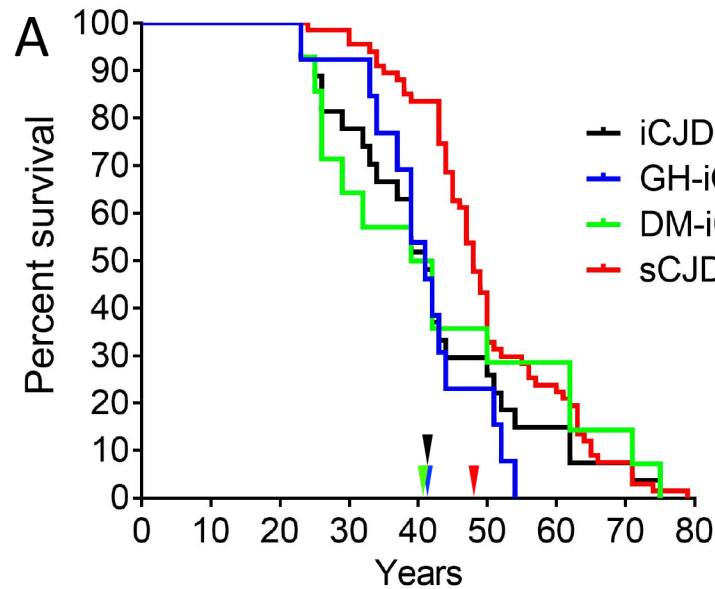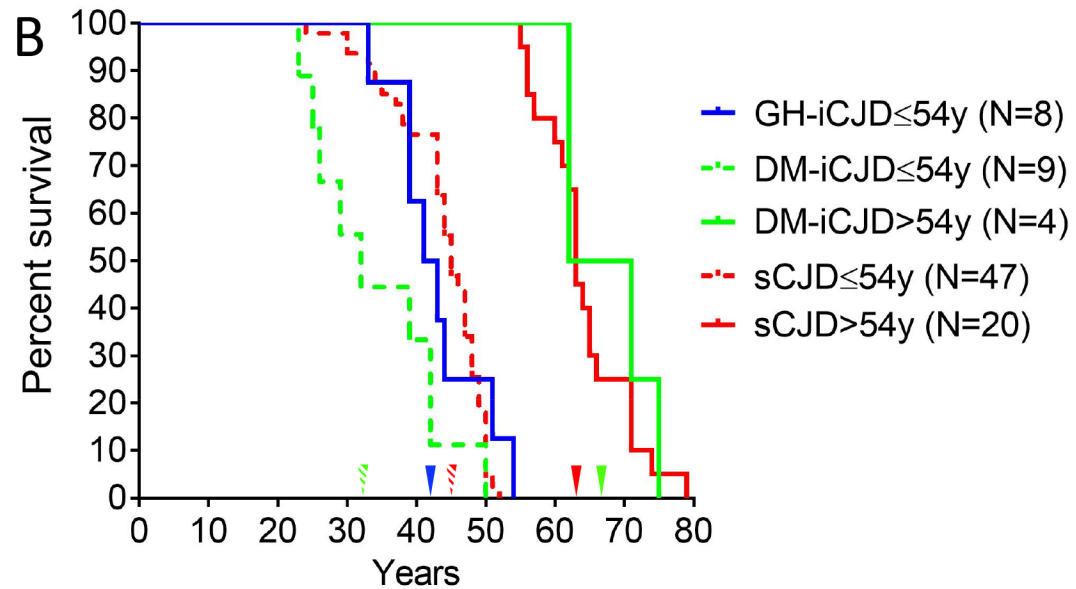

Supplement: Supplementary file 3 — Kaplan–Meier estimates of iCJD and sCJD controls. A: survival graphs representing whole iCJD cohort and its GH- and DM-iCJD subsets as well as sCJD cases. B: survival graphs of 21 iCJD and all sCJD divided into two groups according to age: 54 years or younger (≤54y), and older than 54 years (> 54y). Median survivals are indicated on the X-axis in A and B (arrowheads). Significant differences in median survivals, determined by the log rank (Mantel-Cox), were found between GH-iCJD and sCJD (P=0.0017) (A) or DM-iCJD ≤ 54y and sCJD ≤ 54y (P=0.0027) (B). (PDF 2348 kb) [file 40478_2017_503_MOESM3_ESM.pdf]

Supplementary Figure 2

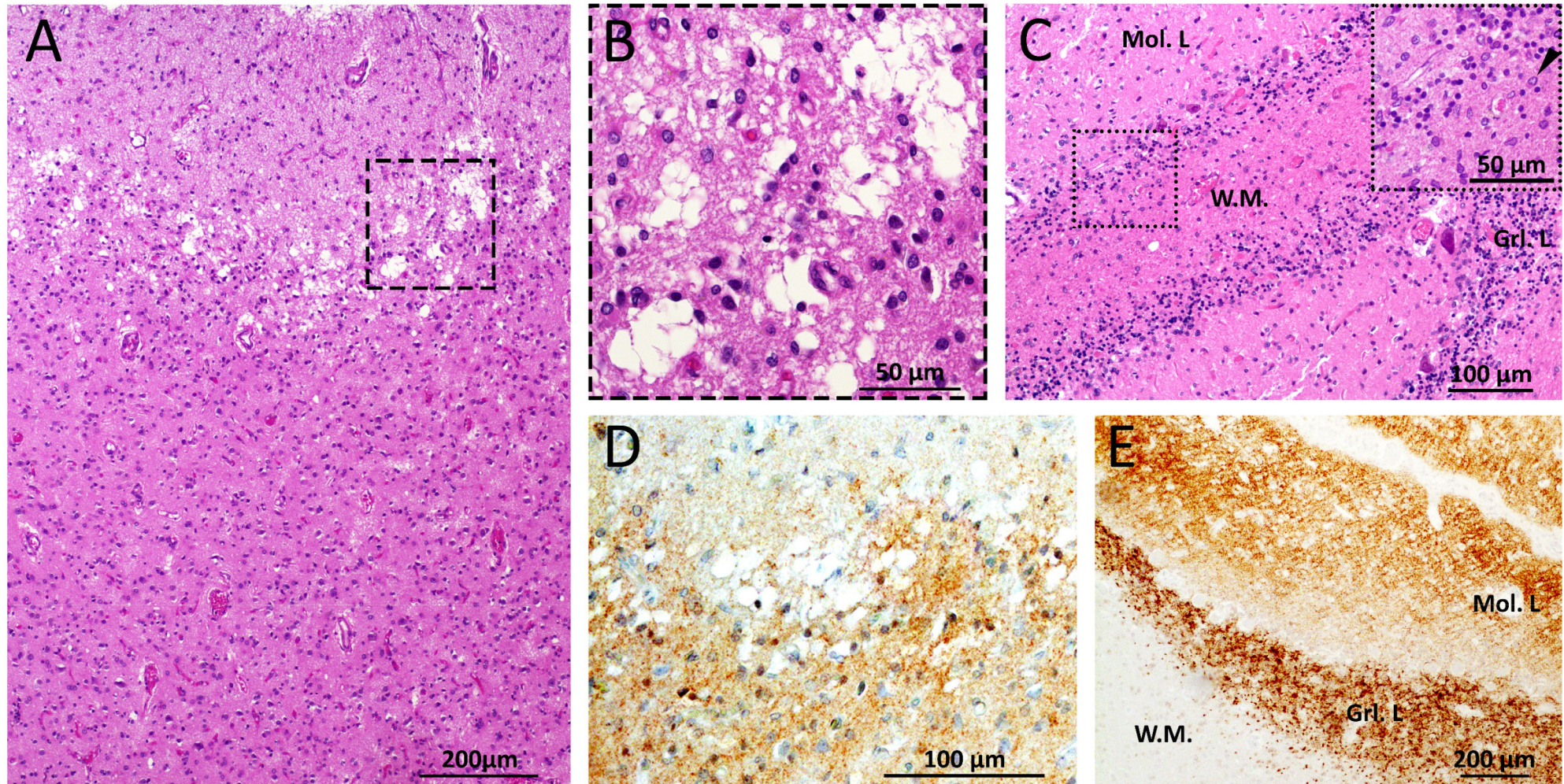

Supplement: Supplementary file 4 — Histopathology of an atypical US iCJD (case 5, Table 1). A-C: HE staining. D and E: PrP immunohistochemistry. A: spongiform degeneration (SD) with large and confluent vacuoles typical of the sCJDMM2 subtype, but limited to layer II of the frontal cortex, and severe neuronal loss and gliosis affecting all layers. B: high magnification of the area highlighted in A showing SD with large vacuoles. C: severe loss of granule cells and gliosis of the cerebellum; Grl. L: granular layer; Mol. L: molecular layer. D and E: diffuse or “synaptic” PrP immunostaining in the frontal cortex (D), and molecular and granular layers of the cerebellum (E); A-C: HE staining. D and E: PrP immunohistochemistry with Ab 3F4. (PDF 30981 kb) [file 40478_2017_503_MOESM4_ESM.pdf]

# Supplementary Figure 3

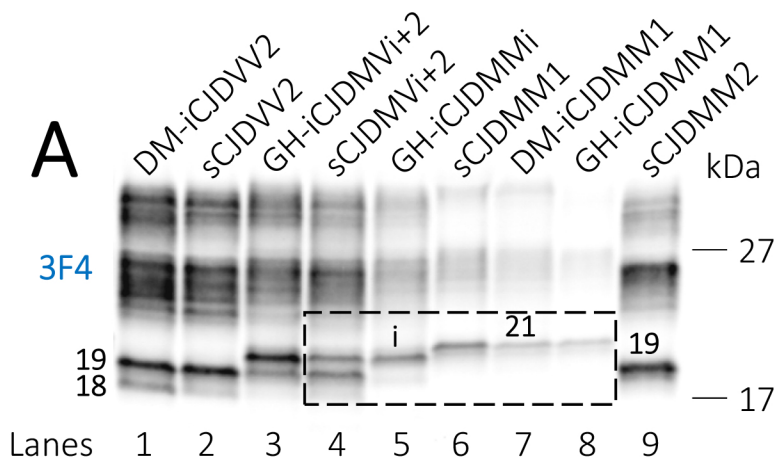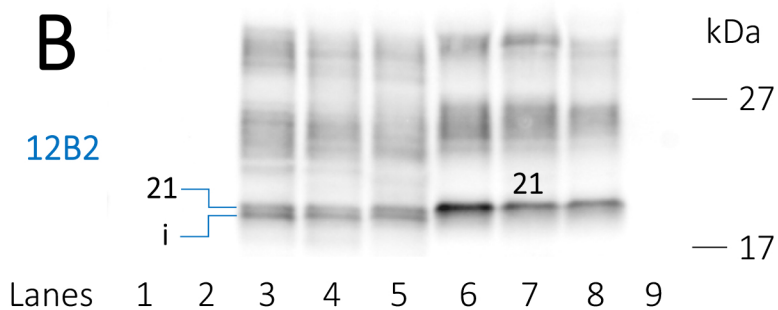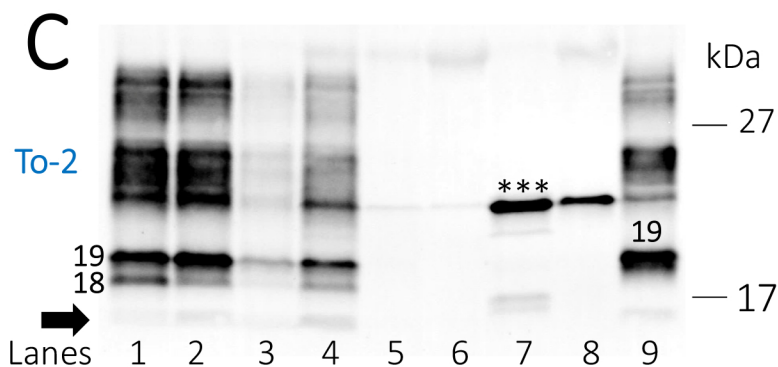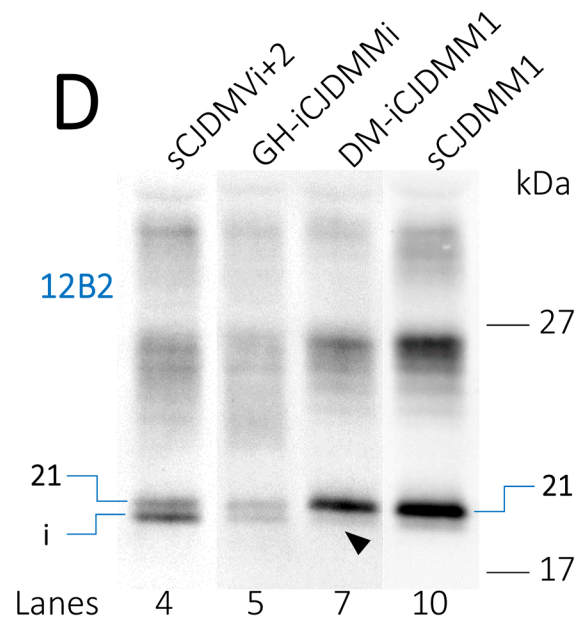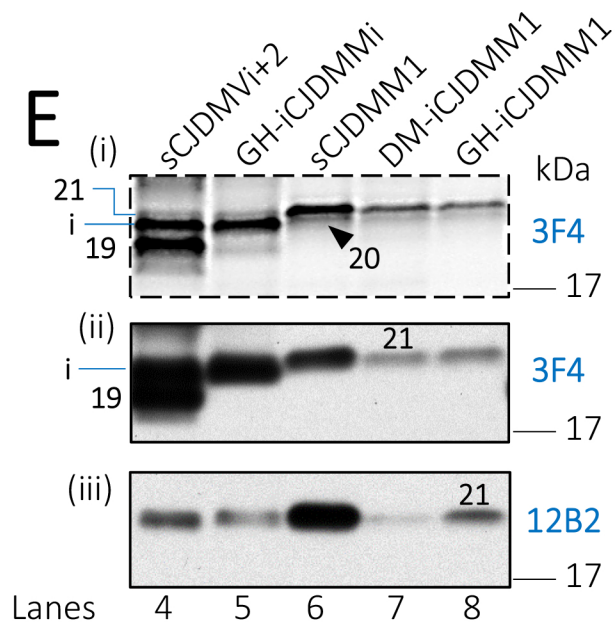

Supplement: Supplementary file 5 — WB profile of resPrPSc from iCJD and sCJD controls using different WB systems for PrP detection. BHs from the frontal cortex (lanes 8-10) and cerebellum (lanes 1-7) treated with 100 U/ml PK (~2000 μg/ml) were probed with indicated Abs. PrP bands were resolved in 15% Tris-HCl, 8.7 cm-long (A-C and E) and 20 cm-long (D) gels, and visualized with near-infrared LI-COR system (A-D and E(i)) or chemiluminescence (E(ii) and E(iii)). A: profiles of resPrPSc from CJD obtained with an 8.7 cm-long gel are similar to those obtained with the 20 cm-long gels in Fig. 2. The unglycosylated resPrPSc ~21 kDa of type 1 (21), ~20 kDa of type i (i), ~19 kDa of type 2 (2) and ~18 kDa (18) are indicated. B: the ~20 kDa of type i (i) and ~21 kDa of type 1 (21) (lanes 3-8), but not the ~19 kDa band of type 2, immunoreact with Ab 12B2. Profile of resPrPSc from iCJDMMi (lane 5) is indistinguishable from that of CJDMVi+2 (lanes 3 and 4), but differs from those of iCJDMM1 and sCJDMM1 (lanes 6-8), as the ~21 kDa band predominates in these three conditions. C: only ~19 kDa (type 2), but not ~21 kDa (type 1) and ~20 kDa (type i) immunoreacted with Tohoku-2 (To-2). An ~18 kDa (18) band in CJDVV2 and CJDMVi+2, and an unidentified lower size fragment (large arrow) in all cases harboring type 2 were also detected. A non-specific band (indicated by three asterisks) was present in all tested samples. D: high resolution gel electrophoresis in 20-cm long gel revealing ~21 kDa (21) and the ~20 kDa band (i and arrowhead). E(i): enhanced image of dashed area in A showing the bands ~21 kDa (21), ~20 kDa (i and arrowhead), and ~19 kDa (19; lane 4). The detection of the resPrPSc ~20 kDa band in addition to the prominent ~21 kDa fragment in CJDMM1 is most likely due to our use of a high resolution electrophoretic systems. E(ii): profiles of unglycosylated resPrPSc from the same cases in E(i) visualized on film by chemiluminescence. Note that the three CJDMM1 (lanes 6-8) show only ~21 kDa bands. E(iii): the ~20 k [file 40478_2017_503_MOESM5_ESM.pdf]

# Supplementary Figure 4

A

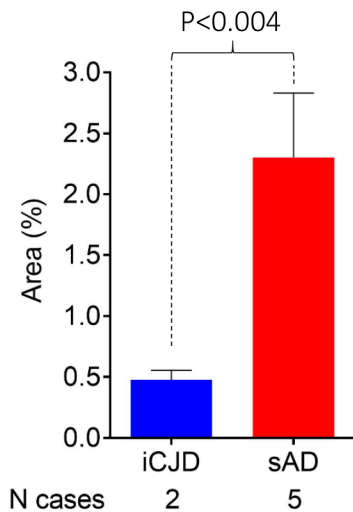

B

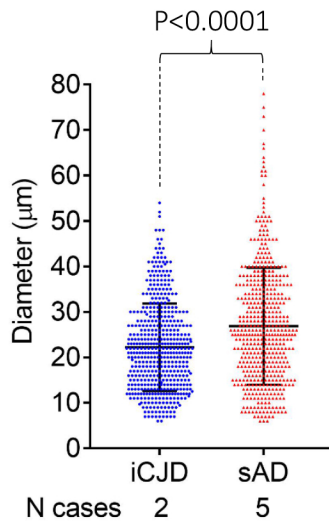

C

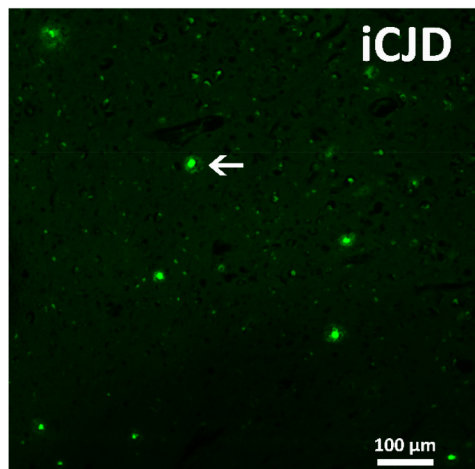

D

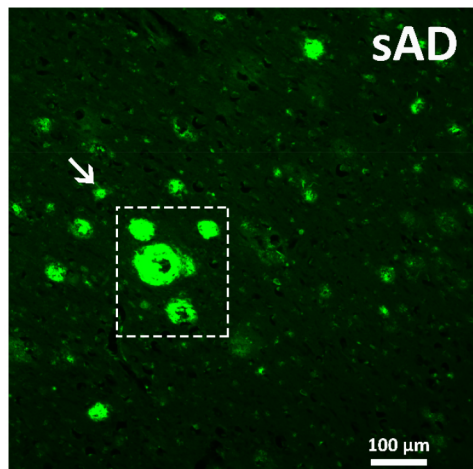

Supplement: Supplementary file 6 — Quantitative estimates of Thioflavin S-positive Aβ CP affecting the cerebral cortex in subjects with iCJD and sporadic AD (sAD). A: the density of Aβ CP, expressed as the percentage of cerebral cortex area occupied, was 5 times greater in subjects with sAD than in iCJD. B: size of Aβ CP (N=500), expressed as diameter, was greater in patients with sAD than in those with iCJD. C and D: representative microscopic fields showing fewer and smaller CP (arrows) in iCJD (C) than sAD (D) where cluster of very large CP (dashed square) could be detected. Bar graphs are expressed as mean ± standard error of the mean (SEM) in A or as mean ± standard deviation in B. Student's t-test (two-tailed). (PDF 3338 kb) [file 40478_2017_503_MOESM6_ESM.pdf]

Supplementary Figure 5

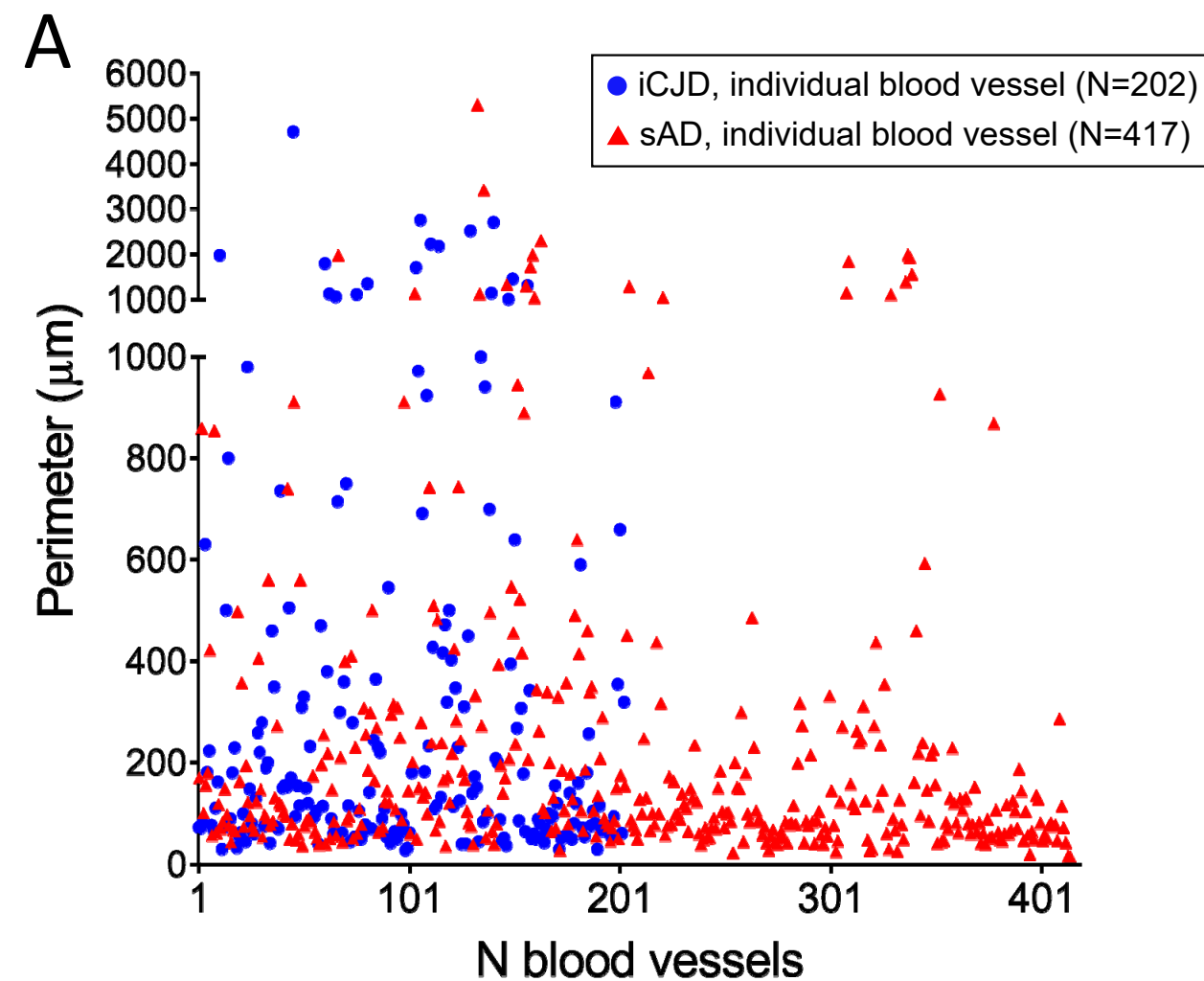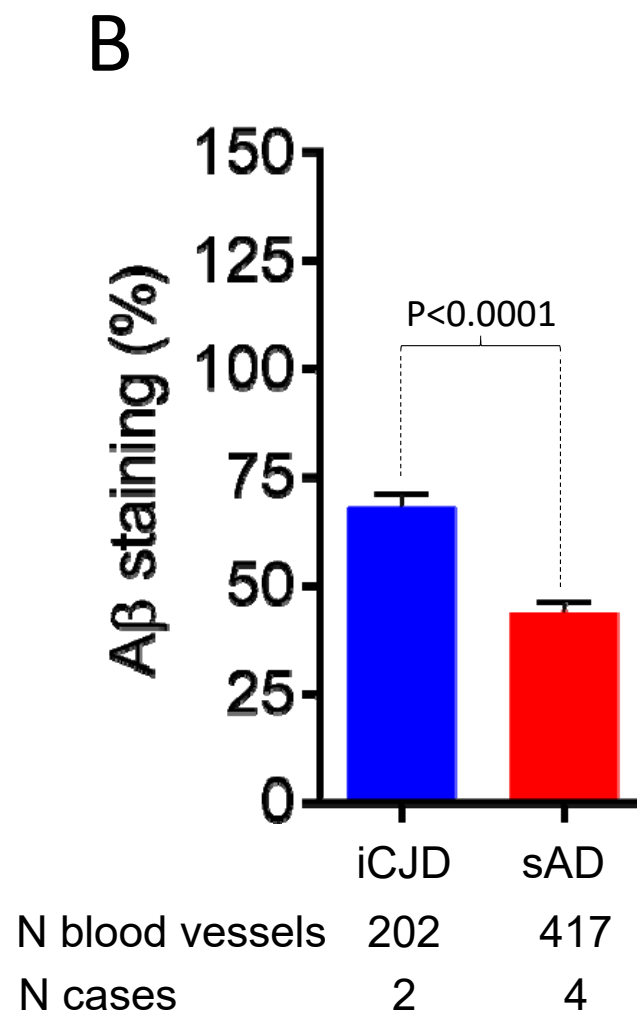

Supplement: Supplementary file 7 — Size of blood vessels examined and semiquantitative evaluation of the percentage of the vessel wall perimeters occupied by Aβ deposits in subjects with iCJD and sporadic AD (sAD). A: scatter plot showing the similar sizes of the blood vessels from the subarachnoid spaces of frontal, occipital and cerebellar regions examined for the semiquantitative determinations made in B; size of individual vessels is measured as perimeter. B: the percentage of the blood vessel wall occupied by Aβ was significantly greater in iCJD than sAD. Bar graphs are expressed as mean±SEM. Student's t-test (two-tailed). (PDF 228 kb) [file 40478_2017_503_MOESM7_ESM.pdf]
